# Large libraries of single-chain trimer peptide-MHCs enable antigen-specific CD8+ T cell discovery and analysis

**DOI:** 10.1038/s42003-023-04899-8

**Published:** 2023-05-16

**Authors:** William Chour, Jongchan Choi, Jingyi Xie, Mary E. Chaffee, Thomas M. Schmitt, Kathryn Finton, Diana C. DeLucia, Alexander M. Xu, Yapeng Su, Daniel G. Chen, Rongyu Zhang, Dan Yuan, Sunga Hong, Alphonsus H. C. Ng, Jonah Z. Butler, Rick A. Edmark, Lesley C. Jones, Kim M. Murray, Songming Peng, Guideng Li, Roland K. Strong, John K. Lee, Jason D. Goldman, Philip D. Greenberg, James R. Heath

**Affiliations:** 1 Institute for Systems Biology, Seattle, WA 98109 USA; 2 Division of Biology and Biological Engineering, California Institute of Technology, Pasadena, CA 91125 USA; 3 Molecular Engineering & Sciences Institute, University of Washington, Seattle, WA 98195 USA; 4 Program in Immunology, Fred Hutchinson Cancer Research Center, Seattle, WA 98109 USA; 5 Division of Human Biology, Fred Hutchinson Cancer Research Center, Seattle, WA 98109 USA; 6 Division of Chemistry and Chemical Engineering, California Institute of Technology, Pasadena, CA 91125 USA; 7 Department of Microbiology and Department of Informatics, University of Washington, Seattle, WA 98195 USA; 8 Department of Bioengineering, University of Washington, Seattle, WA 98195 USA; 9 PACT Pharma, South San Francisco, CA 94080 USA; 10 Institute of Systems Medicine, Chinese Academy of Medical Sciences & Peking Union Medical College, Beijing, 100005 China; 11 Suzhou Institute of Systems Medicine, Suzhou, 215123 China; 12 Key Laboratory of Synthetic Biology Regulatory Element, Chinese Academy of Medical Sciences, Beijing, China; 13 Division of Medical Oncology, Department of Medicine, University of Washington, Seattle, WA 98195 USA; 14 Swedish Center for Research and Innovation, Swedish Medical Center, Seattle, WA 98104 USA; 15 Division of Infectious Disease, Department of Medicine, University of Washington, Seattle, WA 98195 USA; 16 Department of Immunology, University of Washington, Seattle, WA 98195 USA

**Keywords:** Immune cell isolation, Applied immunology

## Abstract

The discovery and characterization of antigen-specific CD8^+^ T cell clonotypes typically involves the labor-intensive synthesis and construction of peptide-MHC tetramers. We adapt single-chain trimer (SCT) technologies into a high throughput platform for pMHC library generation, showing that hundreds can be rapidly prepared across multiple Class I HLA alleles. We use this platform to explore the impact of peptide and SCT template mutations on protein expression yield, thermal stability, and functionality. SCT libraries were an efficient tool for identifying T cells recognizing commonly reported viral epitopes. We then construct SCT libraries to capture SARS-CoV-2 specific CD8^+^ T cells from COVID-19 participants and healthy donors. The immunogenicity of these epitopes is validated by functional assays of T cells with cloned TCRs captured using SCT libraries. These technologies should enable the rapid analyses of peptide-based T cell responses across several contexts, including autoimmunity, cancer, or infectious disease.

## Abstract (web summary)

A high-throughput platform enables the production of single-chain trimers for any pairing of peptide and Class I HLA allele for rapid discovery of epitopes and capture of antigen-specific CD8 + T cells for functional characterization.

## Introduction

The steady emergence of novel, pathogenic virus strains has driven the need for high-throughput approaches for epitope-based reagent production^1^. In particular, peptide-major histocompatibility complex (pMHC) reagents, used to capture antigen-specific T cells and extract relevant T-cell receptor (TCR) genes, are fundamental tools for interrogating immunodominant epitopes in host immune responses. Such reagents are also key for efficiently evaluating emerging vaccines or cell-based therapies that are designed to promote antigen-specific T-cell responses against disease. These therapies are often guided by HLA-based epitope prediction algorithms, which may generate hundreds of putative antigens per HLA allele. To accommodate this scale, there is an outstanding need for libraries of soluble pMHC reagents prepared in a high-throughput manner to identify antigen-specific TCRs from peripheral blood mononuclear cells (PBMCs) from individuals with diverse HLA alleles.

Soluble pMHCs are conventionally produced by expression of the subunits of the MHC within *Escherichia coli*, followed by in vitro refolding of the HLA heavy chain and β2-microglobulin (β2m) subunit inclusion bodies in the presence of a target peptide^2^. A modified version incorporates an ultraviolet (UV) light-cleavable peptide into the pMHC^3–5^, enabling rapid production of pMHC libraries via UV cleavage followed by antigen exchange. Typically, the overall protein yield from such methods is HLA- and peptide-dependent, and reagents produced by either method have a limited shelf-life.

Single-chain trimers (SCTs) provide an alternative pMHC construction that may address these issues^6–8^. Briefly, a pcDNA3.1 plasmid construct encodes the IFNα2 protein secretion signal (MALTFALLVALLVLSCKSSCSVG)^9^, peptide, peptide-β2m linker (L1), β2m, β2m-HLA linker (L2), HLA, and protein purification tags, and the peptide-L1-β2m-L2-HLA construct is secreted as one protein. SCTs were adopted into mammalian expression systems, enabling significant improvements to overall protein yield, presumably due to the use of internal protein folding and quality control mechanisms^8^. SCTs have also been engineered with binding pocket mutations to minimize the functional influence of the peptide linker and to improve the immunogenicity of the pMHC reagents^10–13^. Recently, the SCT system was adapted into Expi293 cells to maximize expression quantity. Peptide modularity was introduced using homologous recombination of peptide-encoded DNA fragments into the plasmid to enable the production and functional characterization of two SCTs encoding HLA-A*24:02 viral peptides^14^. These works point to the potential use of SCTs as functional alternatives for building pMHC libraries, which is the avenue we explore here.

We describe a high-throughput platform enabling the production of SCTs for any pairing of peptide and Class I HLA allele. Whereas pMHC folding, epitope, and HLA modularity are determined by peptide synthesis and refolding of expressed MHC subunits, respectively, the SCT platform utilizes a primer and a PCR template plasmid to determine these two variables. The facile nature of handling and scaling these PCR reagents enables a mix-and-match approach that allows one to rapidly screen across a peptide library and HLA template variants. We first demonstrate a test case of 18 tumor-associated antigens (TAAs) for HLA-A*02:01, utilizing nine different L1/HLA templates to assess the impact of peptide and L1/HLA template on SCT protein expression and thermal stability. Next, we highlight SCT functionality in a disease context by demonstrating SCTs loaded with epitopes derived from common viral strains. We then utilize our SCT platform to enable the assessment of hundreds of viral epitopes, culminating in the discovery of immunodominant epitopes across two SARS-CoV-2 protein domains and the isolation of their cognate TCRs. Finally, we clone and functionally characterize the cytotoxic killing capacity of CD8^+^ T cell clonotypes discovered using the SCT library approach, validating the utility of this strategy.

## Results

### Preparation of SCT libraries

**Fig. 1:**
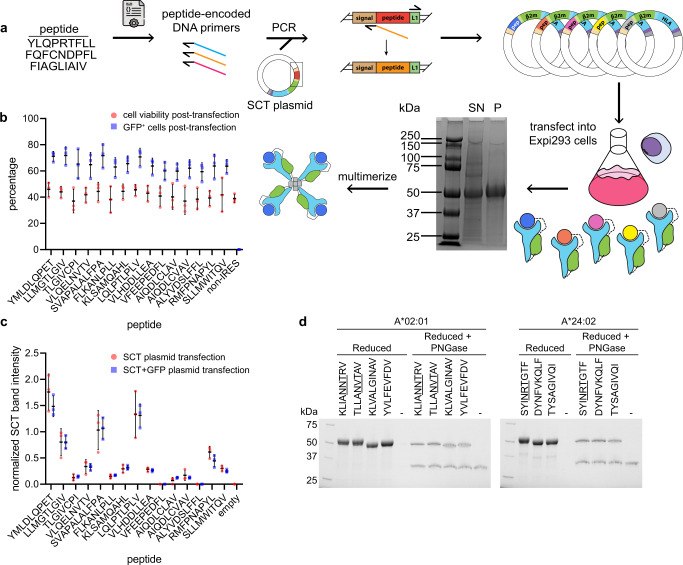
SCT production and quality control. **a** Workflow of SCT production. Reduced SDS-PAGE gel showing expressed SCT protein (~50 kDa); SN supernatant, P His-Tag purified SCT. **b** Cell viability and transfection efficiency of SCT plasmid library. **c** SCT yield from each peptide element of the SCT plasmid library, with or without IRES-GFP reporter. Error bars represent standard deviation, *n* = 3 independent measurements. **d** Reduced SDS-PAGE gel of A*02:01 and A*24:02 SCTs pre- and post-PNGase treatment. NXT glycosylation motifs of peptides are underlined.

The protocol for SCT library preparation is presented in Fig. 1a. A peptide list was first converted into PCR-optimized DNA primers. Inverse PCR of each peptide-encoded primer onto an SCT plasmid template and re-circularization of the product generated each unique peptide variant of a plasmid library. Plasmids were then transfected into Expi293 cells over four days to induce secretion of the SCT protein product. The expressed SCT protein yield was then characterized by a custom Python script for sodium dodecyl sulfate-polyacrylamide gel electrophoresis (SDS-PAGE) gel analysis (Supplementary Fig. 1a–c), followed by biotinylation and His-Tag purification.

To explore the influence of peptide antigen on SCT yield, we prepared a library of 18 SCTs representing known HLA-A*02:01 epitopes (Supplementary Table 1) using a D3 template (see below). The protocol of Fig. 1a was modified to incorporate an IRES-GFP sequence following the SCT region, such that regardless of peptide identity or level of SCT expression, transfected cells would express intracellular GFP^15^ (Fig. 1b, c). Flow cytometry-based detection of GFP-positive cells indicated that transfection efficiency (~70%) was uniform across all tested SCT constructs (Fig. 1b). A biological triplicate of this subset, with and without the IRES-GFP insert, demonstrated consistent SCT yield variations suggesting that the individual peptide epitopes strongly influence the yield of their SCT library elements (Fig. 1c).

The SCTs are expressed in mammalian cells and so may incorporate post-translational modifications that would not be presented in folded pMHCs. We explored this effect by focusing on epitopes that contained the NXT glycosylation consensus sequence. In fact, for SCTs containing such sequences, SDS-PAGE analysis revealed a slightly elevated mass, the origin of which could be confirmed by analyzing the SCTs following de-glycosylation (Fig. 1d). Thus, SCTs can undergo biological protein processing and so have the potential to contain relevant post-translational modifications.

We also compared SCT library yields versus pMHC libraries generated by UV exchange. Starting with A*03:01-restricted putative neoantigens predicted from a melanoma patient^16^, SCTs were assembled using templates D3 and D8 (see a fuller description of these templates below), while the pMHC library was prepared by UV exchange using literature protocols^3,5^. An ELISA assay measuring anti-β2m antibody absorbance was conducted to quantify UV exchange efficiency for each peptide element of the library. A comparison of the SCT yields and UV exchange efficiencies for each peptide (Supplementary Fig. 1d) showed that peptides that lead to high SCT expression generally also exchanged well into UV-pMHCs, and vice-versa.

### Optimizations of SCT template design

**Fig. 2:**
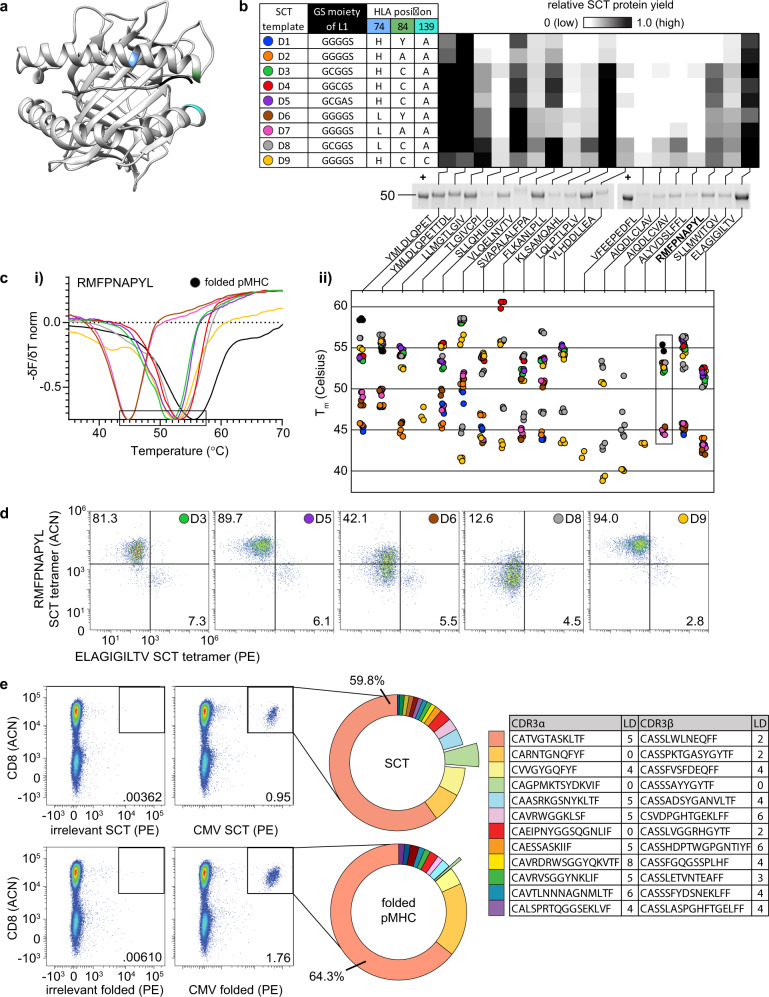
SCT optimization and characterization. **a** Axial view of HLA-A*02:01 SCT crystal structure (RDB ID: 6APN). Highlighted regions of interest: H74 (blue), Y84 (green), A139 (cyan), and the first three amino acids of the L1 linker (black). **b** Table: L1 and HLA amino acid modifications for each SCT template. Heatmap: Relative expression of each SCT combination (*n* = 3), designated by template (row) and peptide (column), and exemplified by reduced SDS-PAGE of 18 SCTs constructed using design template D9. Previously expressed and a purified aliquot of WT1 SCT is used as a positive control (+) for band intensity quantification. **c** Thermal melting profiles of SCTs. The negative of the change in fluorescence over the change in temperature (−*δF*/*δT*) is measured for SCTs encoding WT1 peptide (**c.i**). Local minima representing Tm values (see the boxed region of WT1 plot) are plotted (**c.ii**) for each SCT template and peptide (*n* = 3). **d** WT1 SCTs constructed according to each of the six template designs were paired with a MART-1 SCT (D3 template) to identify cognate TCR-transduced cells. The number/color at the top right of each plot indicates the SCT template used for WT1 SCT tetramer in the flow assay. Percentages indicate the proportion of the total cell population captured in the WT1 SCT-positive quadrant. **e** Capture of CMV-specific T cells using SCT or refolded pMHC format. Unique paired TCR clonotypes identified by 10× single-cell sequencing of tetramer-positive cells. CDR3a and CDR3b sequences of the twelve most frequently captured clonotypes from SCT tetramer along with LD to publicly reported CMV-specific clonotypes from VDJdb are reported in the Table. L1 linker 1, WT1 RMFPNAPYL, MART-1 ELAGIGILTV, CMV NLVPMVATV, LD Levenshtein distance, VDJdb VDJ database, ACN allophycocyanin, PE phycoerythrin.

We next constructed an expanded SCT library with the 18 HLA-A*02:01 epitopes analyzed above and explored the roles that various reported L1/HLA template mutations exerted on both SCT expression yield and SCT performance as an antigen-specific T cell capture agent. Three generations of L1-HLA combinations [closed groove (wild-type HLA), open groove (HLA Y84A), and thiol linker (HLA Y84C)] have been reported as stabilizing (see Fig. 2a for the locations of these mutated residues). We introduced these genetic variants into five unique designs, D1–D9^10–12^ (Fig. 2b). Designs that contain cysteine in the linker (D3–D5) incorporate the HLA Y84C mutation to complete a dithiol linkage. Three templates also contained an H74L mutation^13^ (D6–D8), which forms a portion of the C pocket in the peptide binding groove of the HLA subunit and has been reported to facilitate peptide loading and immunogenicity. Our final design (D9, termed DS-SCT) was inspired by a recent report that the Y84C-A139C mutation in the HLA molecule could introduce further stabilization^17–19^. This 162-element plasmid library (9 HLA templates × 18 peptides) was transfected into Expi293 cells (Fig. 2b). Reduced amounts of the SCT protein bands based on SDS-PAGE analysis were associated with variations in protein yield dependent on peptide and template (Fig. 2b). Templates containing thiol linkers (D3–D9) produced the highest overall yields. For certain peptides, such as AIQDLCLAV and AIQDLCVAV, the strong expression could only be obtained with the D8 template, which incorporates both H74L and thiol linker features.

We explored the thermal stability of this SCT library through thermal shift assays, which utilize differential scanning fluorimetry to measure the intensity of a fluorescent dye (SYPRO orange) that binds to hydrophobic regions of the protein. Less thermally stable proteins exhibit lower melting temperatures. SCTs that were expressed above a yield threshold were HisTag-purified into PBS buffer at pH 7.4. The measured *T*_m_ values were within expected literature ranges^20^ and revealed a trend of increased stability for the same peptide from closed groove to open groove to thiolated linker/groove (Fig. 2c.i–ii). For three peptides (YMLDLQPET, YMLDLQPETTDL, and RMFPNAPYL), folded pMHCs were shown to exhibit a higher relative *T*_m_ than their SCT counterparts (Fig. 2c.ii). Across all peptides, SCT thermal stabilities were also higher for H74L variants than wild-type counterparts. For some peptides (such as AIQDLCLAV) or some template/peptide combinations (such as D7/YMLDLQPET), we detected two distinct melting temperatures. We speculate that the lower temperature arises from an improperly folded SCT, and so we utilize the higher value in Fig. 2c.ii (Supplementary Fig. 2a).

We validated the functionality of the SCT constructs for the Wilms Tumor 1 (WT1) peptide (RMFNAPYL) by assessing tetramer binding of the WT1-specific C4αβ-TCR against select templates (D1, D2, and D7 expression were too low for use) (Fig. 2d)^21^. Expressed WT1 SCTs were purified and then combined with MART1-specific F5 TCR-transduced Jurkat cells in a 95/5 ratio for use in binding assays. We used the MART1 epitope-presenting SCT tetramer (D3 template, Supplementary Fig. 2b) as a stable control. For the WT1 epitope-presenting SCT tetramers, the D3, D5, and D9 templates all yielded excellent performance, selectively capturing 81–94.0% of the WT1-specific cell population (Fig. 2d). Thus, the best tetramer performance did not necessarily correlate with the highest thermal stability. The D8 WT1 SCT design, for instance, comes closest to matching the thermal stability of the folded pMHC but performs poorly. We selected templates D3 and D9 for additional experiments since both exhibited good thermal stability and excellent performance as antigen-specific T-cell capture reagents.

We next compared antigen-specific CD8^+^ T cell capture performance of D3-template SCT multimers and folded pMHC multimers by obtaining sequences of CDR3 regions from TCR α and β chains captured using these reagents. The HLA-A*02:01-restricted CMVpp65 CD8^+^ T cell epitope peptide (NLVPMVATV) was used in interferon (IFN)-gamma ELISPOT assay to identify a CMV-reactive healthy A*02:01 donor for this experiment. This CMVpp65 SCT and its folded pMHC counterpart were multimerized into barcoded dextramers to isolate CMV-specific T cells for 10× single-cell TCR sequencing. A similar distribution of antigen-specific clones was captured by the two reagents (Fig. 2e). Levenshtein distances (LD) of the CDR3α and CDR3β chains against a public database (Fig. 2e, table) indicated high similarity between the detected CMV-specific TCR chains and those previously reported^22^. Two paired clones (red and light orange wedges of Fig. 2e) exactly matched literature CDR3 sequences (LD = 0). An additional clone (light green wedge, Fig. 2e), containing an α/β pair for which both chains have been reported as CMV-specific^23,24^, was captured by the SCT at a tenfold higher frequency relative to the folded pMHC. Thus, SCT tetramers appear to have at least a similar flow cytometry performance to the gold standard of folded pMHCs.

### SCT libraries capture functionally relevant virus-specific T cells

**Fig. 3:**
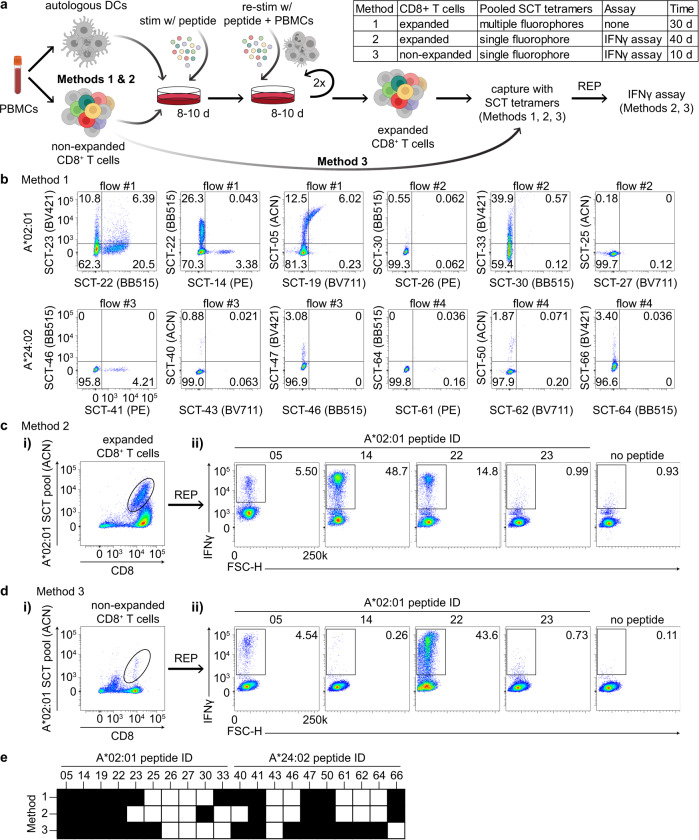
Identification of immunogenic peptides from non-expanded PBMCs using pooled SCT tetramers. **a** Workflow of antigen-specific T-cell identification using SCT tetramers (created with BioRender.com). **b** Representative flow cytometry plots of CD8^+^ T cells captured by 5-color pooled SCT tetramers from peptide-stimulated and expanded CD8^+^ T cells (Method 1) (*n* = 10). **c**, **d** Representative flow cytometry plots of CD8^+^ T cells initially captured by single-color pooled SCT tetramers from peptide-stimulated and expanded CD8^+^ T cells (ci) (Method #2) or from non-stimulated, non-expanded CD8^+^ T cells (**d.i**) (Method #3). Subsequent flow plots represent IFNγ+ cells after peptide stimulation of expanded cells from the previously captured tetramer-positive subset (**c.ii,** **d.ii**). **e**, Binary mapping of peptides which elicit positive signal based upon tetramer binding (Method 1) or IFNγ release (Methods 2 and 3). Values in flow cytometry plots indicate the percentage of the total cell population captured within the quadrant or outlined box. Peptide sequences are found in Supplementary Table 2. REP rapid expansion protocol, BV421 Brilliant Violet 421 nm, BB515 Brilliant Blue 515 nm, PE phycoerythrin, ACN allophycocyanin, BV711 Brilliant Violet 711 nm.

We next explored how SCT libraries might be used to improve and streamline literature protocols for the capture of antigen-specific T cells through the successive exploration of three methods (Fig. 3a–d). We started with an established protocol and then used SCT libraries to streamline and simplify that protocol. Using template D3, we first expressed an SCT library targeting 66 known epitopes from common viral strains (CMV, EBV, influenza, and rotavirus) for A*02:01 and A*24:02 (Supplementary Table 2). We then selected the ten most highly expressed SCTs for each HLA and synthesized the corresponding peptides. These SCT and peptide reagents were then applied across all three methods.

For Method 1 (Fig. 3a), we used a well-established protocol to generate antigen-specific T cell lines specific for the selected peptides^25,26^. Briefly, monocytes were isolated from healthy donor PBMCs (either A*02:01 or A*24:02) and matured into dendritic cells with a cytokine cocktail. Mature DCs were incubated with 1 µg/mL of pooled HLA-restricted peptides and then irradiated. This promotes the presentation of the peptide antigens by these DC cells while also rendering the DC cells non-proliferative. CD8^+^ T cells purified from autologous PBMCs were then incubated with these peptide-loaded DCs for 8–10 days to induce selective stimulation and expansion of antigen-specific CD8^+^ T cells. The T cell lines were twice again stimulated and expanded with peptide-pulsed irradiated autologous PBMCs. For each HLA allele, this process was replicated 10× using separate aliquots of CD8^+^ T cells from the HLA-matched donor. To test these expanded T cell populations for specificity to the 20 peptides, we prepared four pools, each comprised of 5 SCT tetramers conjugated to different fluorochromes. The individual lines from each HLA-matched donor were then analyzed by 5-color flow cytometry (Fig. 3b, see Supplementary Fig. 3a for gating strategy) using these SCT tetramer pools (see Methods). In this way, the flow cytometry assay identifies which antigens promoted clonal expansions and which ones were irrelevant, and it tests for binding selectivity against those relevant antigens. SCT tetramer-positive T cell populations were identified from each line, with little evidence of cross-reactivity across the other relevant and irrelevant SCTs. This indicates that for these subsets of peptides and for each donor, T cell populations exist that selectively bind to peptides presented via antigen-presenting cells (APCs) and also bind to the cognate SCTs (Fig. 3b).

The success of Method 1 suggested the potential for a larger library approach for the isolation and characterization of antigen-specific T cells. Therefore, for Method 2 (Fig. 3a), we explored whether a population of antigen-specific T cells with a broad diversity of antigen specificities could be identified from a polyclonal T cell pool when tetrameric SCTs were pooled together into a library format. We first assessed Method 2 using the antigen-enriched T cell lines described above (Fig. 3b), where the breadth of available antigen specificities was known. All T cell lines from each donor were pooled and stained with a pooled library of SCT tetramers, where all 10 SCTs were conjugated to allophycocyanin (ACN) dye and then combined as a single staining solution. The sample was sorted for ACN-positive T cells (Fig. 3c.i), which were then expanded using a T cell rapid expansion protocol (REP)^27^. To assess the frequency of T cells with distinct antigen-specificities within this population, we assessed IFNγ production by the expanded cells in response to each peptide (Fig. 3c.ii, see Supplementary Fig. 3b for gating strategy). The responding cells contained T cell specificities that closely matched those found in Method 1 (Fig. 3e), confirming that T cells that bind each of the tested SCTs are, in fact, reactive against the native peptide. Thus, Methods 1 and 2 show that SCTs may be pooled together to capture and promote the expansion of targeted antigen-specific T-cell populations.

For Method 3 (Fig. 3a), we asked whether we could use the same library of SCTs to purify similar populations of T cells from *unmanipulated* CD8^+^ T cells ex vivo isolated from the donor PBMCs. This streamlined approach can circumvent the 20 days of PBMC-facilitated peptide stimulation and expansion of Methods 1 and 2. We prepared ACN-conjugated SCT tetramers in a pooled, single-fluorophore format (identical to Method 2) to directly sort (Fig. 3d.i) non-expanded CD8^+^ T cells from the same donors. T cells were then expanded using the same REP (Fig. 3a). Figure 3d.ii shows the measurements of IFNγ secretion of tetramer-sorted and expanded CD8^+^ T cells from A*02:01 donor PBMCs upon individual peptide stimulation. Additional data is provided in Supplementary Fig. 3c–e for the other A*02:01 and A*24:02 peptides. Notably, the epitopes for which T cells could be isolated were very similar across all three methods (Fig. 3e). Variations in the frequency of some epitope-specific TCRs were observed, likely reflecting differences in peptide affinity and/or in vitro expansion. Method 3 thus demonstrates that the clonal repertoire in terms of peptide-reactivity could be recapitulated using a pooled SCT and streamlined expansion protocol. This highly efficient method should also reduce expansion bias against the native TCR repertoire.

The above studies were done with well-characterized immunodominant viral epitopes. We next assessed whether this approach could be effectively used to evaluate predicted epitopes from a less characterized pathogen.

### SCT libraries enable the rapid discovery of immunodominant SARS-CoV-2 epitopes

To enumerate the epitope landscape of SARS-CoV-2-specific CD8^+^ T cells, we generated SCTs encoding putative antigens. The NetMHC4.0 binding prediction algorithm was used to identify 9- to 11-mer peptide sequences from the spike protein with 500 nM or stronger binding affinity^28^ to either HLA-A*02:01, A*24:02, or B*07:02. We identified 96, 51, and 33 peptides for these alleles, respectively, with some overlap against published lists of putative antigens^29–31^. The A*02:01 SCTs exhibited useable levels of expression for epitopes throughout the protein except for the trans-membrane (TM) region, which had uniformly weak expression (Supplementary Table 3). B*07:02 SCT expression showed a preference for the N-terminal domain (NTD), the S1/S2 cleavage site, and parts of the S2 subunit (Supplementary Table 4), while highly expressed A*24:02 SCTs were concentrated around the NTD, the receptor binding domain (RBD), and TM regions (Supplementary Table 5). The same prediction process was performed for the Nsp3 protein (papain-like protease, PLpro) for A*02:01 to produce 191 peptides (Supplementary Table 6). All SCTs were generated using the D9 template (Fig. 2b). The B*07:02 SCT library was also expressed using the D3 template. While most SCTs expressed using the D9 template were also expressed with the D3 template (and vice versa), the expression levels were generally higher for D9 (Supplementary Fig. 4a, b), perhaps suggesting that D9 is a superior template. The most strongly expressed SCTs (see Methods) were assembled into four libraries.

**Fig. 4:**
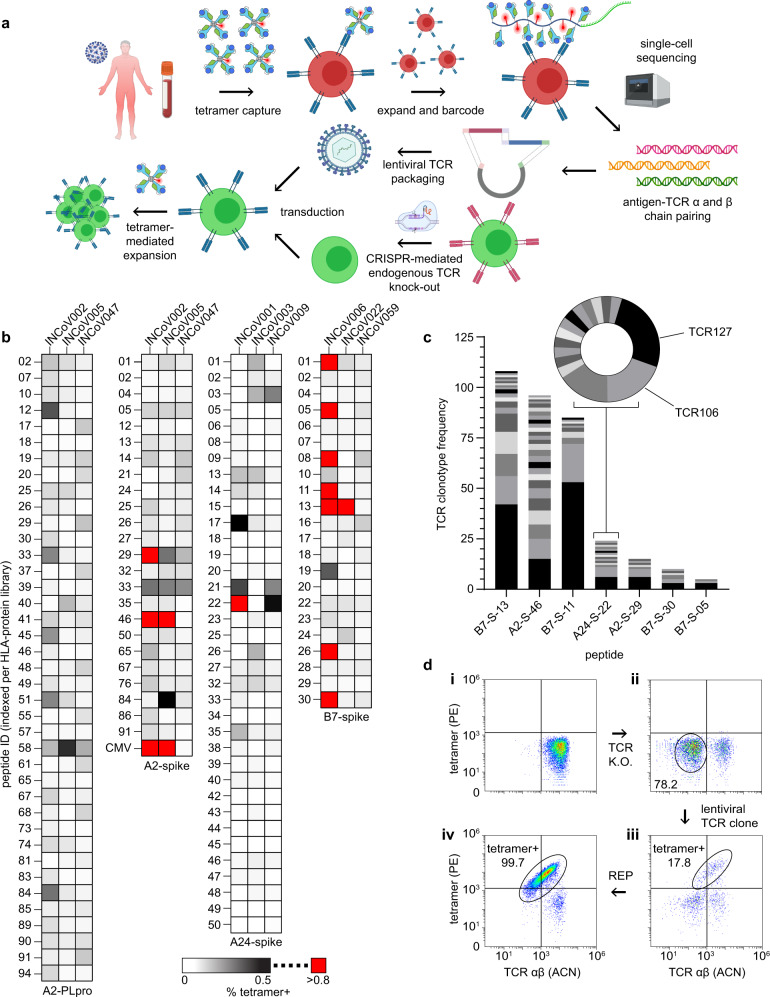
Isolation and cloning of TCRs specific to SARS-CoV-2 epitopes across three Class I HLA alleles. **a** Workflow for SCT-facilitated capture of SARS-CoV-2 antigen-specific CD8^+^ T cells, single-cell TCR sequencing, and TCR cloning into autologous T cells (created with BioRender.com). **b** SCT tetramer-positive CD8^+^ T cells from HLA-matched COVID participants for SCT libraries (*n* = 1). **c** Frequency of unique TCR clonotypes against peptides whose SCTs produced high % tetramer binding (red boxes of (**b**)). **d** Representative flow cytometry plots of T cell cloning workflow. Autologous T cells (**d.i**), T cells after CRISPR-mediated TCR knockout (**d.ii**), tetramer+ T cells after lentiviral TCR cloning (**d.iii**), tetramer+ T cells after sort and REP (**d.iv**). Peptide sequences are found in Supplementary Tables 3–6. A2 A*02:01, A24 A*24:02, B7 B*07:02, PLpro papain-like protease, P PLpro, S spike, PE phycoerythrin, ACN allophycocyanin, REP rapid expansion protocol.

We first used these libraries to ask whether immunogenic epitopes were shared among HLA-matched COVID-19 participants. SARS-CoV-2 SCTs from each library were assembled into PE-tetramer reagents and pooled to stain and sort for antigen-specific T cells from PBMCs collected from three participants per HLA haplotype (Fig. 4a), plus PBMCs from a (never infected) A*02:01 healthy donor. An A*02:01 SCT expressing the CMV pp56 peptide was conjugated with ACN-streptavidin and served as a control. Captured T cells were then expanded using the REP, and antigen specificity was confirmed by flow cytometry with individual SCT tetramers (Fig. 4b). SARS-CoV-2-specific CD8^+^ T cells against the same epitopes were detected in different participants (Fig. 4b), suggesting that, for a given HLA haplotype, immunodominant epitopes are present, consistent with other reports^32–38^. In fact, certain antigen-specific T-cell responses detected here (against epitopes QYIKWPWYI, NYNYLYRLF, SPRRARSVA, and YLQPRTFLL, although YLQPRTFFK was found here to be more immunodominant), have been reported elsewhere^35,37–39^. Note that CMV-specific T cell populations were initially detected in the unexpanded PBMCs from the two A*02:01 COVID-19 participants (1.22% and 0.24%) and detected at 13.2% and 7% after T cell expansion, respectively, again indicating that antigen-specific T cells resting in blood could be successfully captured and expanded using SCT tetramers (Supplementary Fig. 4c.i–iv).

To probe the TCR repertoire of the SARS-CoV-2-specific T cells, selected expanded populations were stained with SCT-loaded DNA-barcoded dCODE dextramers (Immudex) to enable pairing of the SCT capture reagent with specific TCR clonotypes through 10× single-cell sequencing (Fig. 4a). For 7 SCTs representing 7 antigens and 3 HLA alleles, we identified a predominant clonotype (Fig. 4c) and many subdominant clonotypes. Several of these clonotypes were selected for cloning.

Primary CD8^+^ T cells from HLA-matched healthy donors were used to clone putative SARS-CoV-2 antigen-specific TCRs. Flow cytometry (Fig. 4d) was used to monitor key steps in the cloning process (see Methods), including CRISPR/Cas9 knockout of the endogenous TCR α/β chains^40^ (Fig. 4d.i, ii). SCT tetramers were used to assess both the effectiveness of the lentiviral transduction of new TCR α/β genes (Fig. 4d.iii) as well as the purity of the subsequently expanded TCR-engineered cells (Fig. 4d.iv). Using this process, we prepared 31T cell clonotypes representing specificities against 13 different SARS-CoV-2 antigens presented by 3 HLA alleles, plus 2 positive controls.

### Functional characterization of SARS-CoV-2 antigen-specific CD8+ T cells identified using SCT libraries

**Fig. 5:**
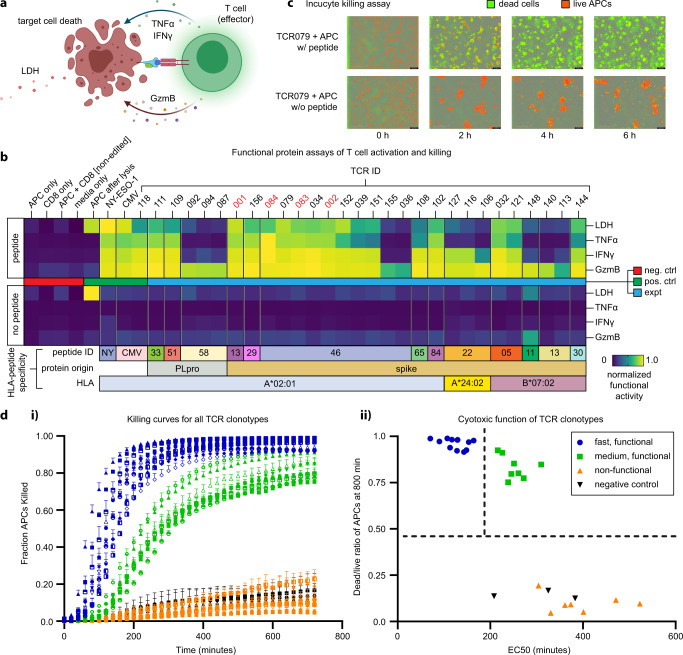
Functional characterization of SARS-CoV-2-specific TCRs. **a** Illustration of functional assays, including T cell-secreted markers of activation and cytotoxicity following antigen-specific activation, as well as the release of LDH following T cell killing of APCs (created with BioRender.com). **b** Heatmap representing measured levels of secreted or released proteins from T cell clonotypes with or without peptide stimulation (*n* = 2). TCRs indicated in red font were identified from healthy donors. **c** IncuCyte live-cell imaging of peptide stimulation and cell-killing activity. Scale bars in each panel represent 400 μm. **d.i**. Measurements of the fraction of dead APCs from IncuCyte kinetic imaging analysis of the TCR-engineered T cell clonotypes co-cultured with APCs. Error bars represent standard deviation from independent triplicate measurements. **d.ii**. Classification of T cell clonotypes by killing activities. TCR sequences are found in Supplementary Table 7. LDH lactate dehydrogenase, TNFα tumor necrosis factor α, IFNγ interferon γ, GzmB granzyme B, NY SLLMWITQC (NY-ESO-1), CMV NLVPMVATV, A2 A*02:01, B7 B*07:02, APC antigen-presenting cell, P PLpro, S spike.

We performed functional assays to confirm that TCR-engineered T cells were responsive to stimulation with the target peptide (Fig. 5a). T cells engineered with SARS-CoV-2-specific TCRs were co-cultured with HLA-matched APCs^41^ at an effector-to-target ratio of 2:1 with and without peptide loading (1 µM). We included A*02:01/NY-ESO-1_157–165_ and A*02:01/CMV pp56_495–503_ antigen-specific TCR-engineered CD8^+^ T cells as positive controls.

Following 16 h of co-culture, we assayed the supernatant for three effector molecules (TNF-α, IFN-γ, and Granzyme B) that would be secreted from the TCR-transduced primary CD8^+^ T cells, as well as lactate dehydrogenase (LDH), which is released from lysed target cells. All proteins were measured by ELISA, except LDH, which was assessed using a standard non-radioactive cytotoxicity assay (see Methods). Measurement results are separately normalized by the highest value in each readout and plotted on a heat map. All the negative controls and all TCRs co-cultured with APCs in the absence of target peptide (but at the same DMSO concentration) produced non-detectable levels of functional proteins as well as LDH (Fig. 5b). By contrast, most SARS-CoV-2 specific T cells (20/31), as well as positive controls NY-ESO-1_157–165_ and CMV pp56_495–503_ specific T-cells secreted detectable levels of effector molecules and promoted apoptosis following activation with APC loaded with the correct peptide (Fig. 5b). All SARS-CoV-2 specific T cell clonotypes produced levels of Granzyme B above that of the negative controls, while subsets produced TNF-α and IFN-γ. Positive correlations were found between all three effector molecules and the assayed LDH levels (Supplementary Fig. 5a–c) (TNF-α: *r* = 0.91, *p* < 0.0001; IFN-γ: *r* = 0.87, *p* < 0.0001; Granzyme B: *r* = 0.67, *p* < 0.0001).

Target killing by T cells expressing TCR-079 was assessed with APCs loaded with or without 1 µM cognate peptide (RLITGRLQSL) in an Incucyte live-cell imaging assay with a caspase activation reporter (Fig. 5c). For this assay, we labeled the APCs with live-cell dye (red). These cells were then co-cultured with the engineered CD8^+^ T cells (unlabeled) and with a reagent that captures caspase 3/7 (green) activation. Fluorescent images were collected every 20 min for 12 h. Quantification of green/red fluorescent areas at each time point provided the metric for tracking the kinetics of target cell killing. For this TCR/APC combination, most cell killing events occurred within 4 h, leading to nearly complete eradication of the APCs. This analysis revealed a broad spectrum of cell-killing activity across the TCR-engineered clonotypes (Fig. 5d.i) and a positive correlation with the LDH measurement assay (Supplementary Fig. 5d).

The time course killing curves (Fig. 5d.i) were fitted to sigmoidal functions (see Methods) to extract two comparative killing metrics for assessing the differences between the TCR clonotypes. The first metric was the EC_50_ value, which is the point of the steepest slope of the sigmoidal fit to the killing curve, and the second metric was the dead/live cell ratio at 12 h (Supplementary Fig. 5e). This analysis defined three TCR groups (Fig. 5d.ii). T cells expressing group 1 TCRs (blue curves in Fig. 5d.i and blue circles in Fig. 5.d.ii) were labeled ‘fast and functional.’ These cells exhibited a fast killing response (EC_50_ < 200 min) with a dead/live cell fraction >0.9. Group 2 (green; slow and functional) exhibited an EC_50_ > 350 min but a similar dead/live cell fraction >0.75 as Group 1. Group 3 TCRs (orange, non-functional) exhibited a poor killing response. A full list of TCRs, their cognate antigens, and their groupings is provided in Supplementary Table 7. Note that the NY-ESO-1_157–165_ and CMV pp56_495–503_ positive controls were classified as ‘functional.’ This data shows that antigen-paired TCRs identified through the protocol of Fig. 4a reflect the full spectrum of antigen-specific T cell responses, including a large fraction of highly potent TCRs.

## Discussion

The SCT library technology introduced here permits the assembly of hundreds of pMHCs in a relatively rapid and facile manner. These libraries, in turn, enable highly multiplex searches for antigen-specific CD8^+^ T cell populations. Characterization of SCT libraries revealed distinctive peptide-dependent trends in SCT expression, thermal stability, and functionality. Some peptides have proven difficult to express in the SCT format, and it is possible that this issue results from our expression platform reflecting in part the natural binding affinities of these peptides for the HLA binding groove, as noted by others^8^, and thus may be used as a proxy to validate peptide binding algorithms. It is also possible, of course, that certain peptides that are presented by MHC in the natural, in vivo biological format are not amenable to preparation as an SCT, although this limitation can also apply to in vitro folded pMHCs. Our finding that biologically produced SCTs can contain glycosylated antigens may be notable. Such antigens, especially glycosylated self-antigens in the context of autoimmune disease, are a topic of recent literature^42^. Whether the SCT library technology can provide a new tool for exploring this biology is an open question.

For the HLA A*02:01 allele, we systematically explored how various previously reported mutations in the L1 and HLA domains influenced SCT expression and function. We identified 3 templates (D3, D5, and D9) that yielded nearly identical expression patterns across 19 different viral and tumor-associated antigens. When expressed with the WT1 antigen, they all also yielded selective and efficient capture of C4αβ TCR-engineered WT1 antigen-specific T cells. Each of these templates contained two cysteines to provide a disulfide bridge to promote SCT stability and preserve the wild-type H74 amino acid instead of using the H74L to alter epitope presentation. The HLA-A*02:01-restricted CMVpp65 CD8^+^ T cell epitope expressed with the D3 SCT template was further shown to capture a similar spectrum of T cell clonotypes as the corresponding folded pMHC. Thus, although the folded pMHC may represent the natural conformation, multiple SCT templates appear to adequately mimic this natural conformation with respect to TCR binding and also provide significant advantages in terms of enabling library preparation and providing long-term stability. A ‘best’ template may be dependent upon the HLA allele. For example, for a library of B*07:02-restricted epitopes, we found that the D9 and D3 templates yielded highly correlated expression patterns, but the overall expression from the D9 templates was superior.

The ability to construct large libraries of SCTs across multiple HLA alleles is fundamentally enabling for both the large-scale quantitation of antigen-specific T cell responses and for the genetic and functional characterization of those identified TCR clonotypes. This was demonstrated through the construction and use of large Class I-restricted SARS-CoV-2 SCT libraries for HLA-A*02:01, HLA-A*24:02, and HLA-B*07:02. Further, large SCT libraries across many additional HLA A, B, and C alleles were reported recently by some of us to identify TCRs that were translated into the clinic for adoptive cell transfer cancer immunotherapy^43^. That paper was primarily a clinical study that only briefly described the SCT library technique, but it does offer further evidence of the versatility of the method. We identified that certain previously reported epitope-specific CD8^+^ T cells were detected within PBMCs collected from COVID-19 participants^32–38^ but at low frequencies. Notably, we also detected several shared epitopes that had only been previously suggested as T-cell epitope vaccine candidates based on peptide MHC binding predictions^44–46^. The antigens tested here were based on those predicted by NetMHC 4.0. Improved prediction approaches, based upon the use of multiple algorithms, have been reported^16^, and could be adapted for more comprehensive searches of antigen-specific T cell populations. Thus, the SCT library approach can permit large-scale searches across a viral proteome to quantitate antigen-specific T-cell responses and may provide an enabling tool for T-cell vaccine design.

Functional testing of the antigen-paired TCR clonotypes revealed that SCT libraries could be used to capture T cells exhibiting a broad range of TCR-dependent cytotoxic responses against antigen-presenting cells. While all TCR clonotypes were selectively activated following antigen exposure, that level of activation was diverse. Such diverse responses have been previously reported^47–49^. Mechanistic studies have revealed that TCR-pMHC binding affinity may be one influencing factor but that there are likely others^50–52^. The high-throughput nature of the SCT library approach should provide a powerful new tool for developing a complete metric. Whether such a metric can be applied uniformly or whether it will vary among viral antigens, tumor neoantigens, cancer-testis antigens, etc., is an open question but one which can be addressed using the methods described here.

## Methods

### COVID-19 and healthy individuals

The INCOV cohort included 209 SARS-CoV-2 patients (50% females, aged between 18 and 89 years with an average of 56 years), an expansion on the cohort previously published on acute infection^53^. Potential participants were identified at five hospitals of the Swedish Medical Center and affiliated clinics located in the Puget Sound region near Seattle, WA. All enrolled patients provided written, in-person informed consent. Healthy control samples were obtained from Bloodworks Northwest (Seattle, WA). The research followed the protocol approved by the Institutional Review Board (IRB) at Providence St. Joseph Health with IRB study number STUDY2020000175 and the Western Institutional Review Board (WIRB) with IRB study number 20170658.

#### SCT template production

Class I SCT-encoded plasmids were constructed using a combination of Gibson assembly and restriction enzyme digest methods for insertion into pcDNA3.1 Zeo(+) plasmid (Thermo Fisher Scientific) (Fig. 1a). Briefly, the SCT inserts were designed to be modular to allow for any choice of L1 to be paired with any choice of HLA allele. Because β2m has no allelic variation in the human species, the SCT was split into two Gibson assembly fragments within the β2m region to allow for the decoupling of L1 from HLA. Fragments were purchased from Twist Bioscience, PCR-amplified with KOD HotStart Hi-Fi polymerase (MilliporeSigma), and joined together by Gibson assembly using NEBuilder HiFi DNA Assembly Master Mix (New England Biolabs). The PCR-amplified Gibson product’s flanking regions were digested by EcoRI and XhoI (New England Biolabs) to be ligated into pcDNA3.1’s MCS region at the same enzyme recognition sites. Codon optimization was applied to the designed fragments under three considerations: (1) selection of only highly prevalent codons in the human species, (2) avoidance of continuous gene segments (24+ bp) where GC content is above 60% (to avoid manufacturer error rates during synthesis), and (3) avoidance of key recognition cut sites within the fragments, which must only exist at the flanks of the Gibson product for insertion into pcDNA vector. Subsequently, the design of the second fragment (encoding HLA allele) was automated with a Python script, encompassing all aforementioned design criteria and accounting for all alleles from Class I HLA-A, B, and C loci. The protein sequences of each HLA allele were obtained from an FTP server hosted by The Immuno Polymorphism Database (ftp://ftp.ebi.ac.uk/pub/databases/ipd/imgt/hla/fasta/).

#### SCT peptide library production

A PCR-facilitated approach was implemented to enable the high-throughput substitution of peptides into SCT-encoded plasmids. Briefly, for any given peptide substitution, a peptide-encoded reverse primer (binding to the IFNα2 signal sequence upstream of the peptide region) and a forward primer (binding to the L1 sequence downstream of the peptide region) are required. Both primers have 16 bp annealing regions (reverse primer: 5′-GCCAACAGAACAGCTG-3′, forward primer: 5′-GGTTGTGGAGGTTCTG-3′). The peptide-encoded reverse primer varies for any given peptide, while the forward primer remains fixed across all peptide elements (unless one chooses to use a different L1/HLA template plasmid). Thus, the peptide-encoded reverse primer will typically be extended by another 27–33 bp to account for the insertion of a 9–11 amino acid peptide (e.g., reverse primer for insertion of the peptide YLQPRTFLL: 5′-CAGCAGGAAGGTTCTAGGCTGCAGGTAGCCAACAGAACAGCTG-3′). Extension PCR was conducted with KOD Hot Start polymerase (MilliporeSigma). The product was phosphorylated and ligated with a mixture of T4 Polynucleotide Kinase and T4 DNA Ligase, and then template DNA was digested with DpnI (New England Biolabs). The peptide-substituted plasmids were then transformed into One Shot TOP10 Chemically Competent *E. coli* (Thermo Fisher Scientific). Plasmids were verified by Sanger sequencing using a Python script prior to use in transfection.

#### SCT expression

Purified SCT plasmids were transfected into Expi293 cells (Thermo Fisher Scientific) within 24-well (2.5 ml capacity) plates. Briefly, 1.25 µg of plasmid was mixed with 75 µl Opti-MEM reduced serum media. 7.5 µl of ExpiFectamine Reagent was mixed with 70 µl Opti-MEM reduced serum media, incubated at room temperature for 5 min, and combined with the plasmid mixture. After a 15-minute room temperature incubation, the solution was added to 1.25 ml of Expi293 cells at 3 million cells/ml into a 24-well plate, which was then shaken at 225 RPM at 37 °C in 8% CO_2_ overnight. Twenty hours later, a solution containing 7.5 µl of ExpiFectamine Transfection Enhancer 1 and 75 µl of ExpiFectamine Transfection Enhancer 2 was added to each well. The plate was kept on the shaker using the aforementioned settings for a total of 4 days from the start of transfection. The supernatant of the transfection solution was collected and filtered through 0.22 µm PVFD membrane syringe filters (MilliporeSigma) prior to yielding analysis via SDS-PAGE. The supernatant solutions of SCTs which expressed at high yield, were concentrated down to 200 µl PBS using 30 kDa centrifugal filter units (Amicon) and subsequently biotinylated with BirA enzyme kit (Avidity) overnight. The biotinylated SCTs were then purified with HisTag resin tips (Phynexus) and desalted back into PBS buffer with Zeba 7 K MWCO spin desalting columns (Thermo Fisher Scientific). For long-term storage, the SCTs were re-suspended into 20% glycerol w/v prior to storage at −20 °C.

#### SCT yield characterization

After 4 days of transfection, a 15 µl solution containing a 3:1 mix of transfection supernatant and Laemmli buffer with 10% b-mercaptoethanol was denatured at 100 ◦C for 10 min and subsequently loaded into Bio-Rad Stain-Free gels for SDS-PAGE (200 V, 30 min). A reduced, purified WT1 (RMFPNAPYL) A*02:01 SCT sample in 20% glycerol PBS solution (containing approximately 2 µg) was run in each gel to serve as a positive control and intensity reference for relative protein yield calculation. Images were obtained using a Bio-Rad ChemiDoc MP gel imaging system (manual settings: 45 s UV activation, 0.5 s exposure). A custom Python script was developed for the analysis of SCT proteins run on Stain-Free gels (Bio-Rad). The script allows for a user-defined selection of protein bands of interest and provides background reduction and uniform normalization of SCT yield across all gels, given the consistent use of a control protein lane. The accuracy of this approach was measured by SDS-PAGE of titrated, pre-quantified samples of purified SCTs to demonstrate a 99% correlation between true protein A280 concentration (as measured by NanoDrop 8000 Spectrophotometer) and quantified relative band intensity (Supplementary Fig. 1). SCTs which expressed above an established cutoff for yield (>0.15 relative intensity to WT1 SCT positive control standard) were selected for subsequent biotinylation and purification steps.

#### Thermal stability characterization

SYPRO™ Orange Protein Gel Stain was purchased from ThermoFisher Scientific and diluted with H2O to give a 100X working solution. To each 19 µl aliquot of Class II SCT protein solution (diluted to 10 µM, if possible), 1 µl of the 100× dye solution was added. A Bio-Rad thermal cycler equipped with a CFX96 real-time PCR detection system was used in combination with Precision Melt Analysis software to obtain the melting curves of each SCT sample. Thermal ramp settings were 25 °C to 95 °C, 0.2 °C per 30 s.

#### Production of tetramers from SCTs

Tetramers were generated by combining monomer SCT and fluorophore-conjugated streptavidin (Thermo Fisher Scientific) at a 4:1 molar ratio in PBS to give a final SCT tetramer concentration of 2 µM (with regard to the SCT monomer). An excess of unlabeled biotin was added to block the free biotin binding site on streptavidin. Following assembly, 20 nM of tetramer (with respect to the SCT monomer) was used to stain up to 1 ×10^6^ cells. Tetramers were stored at 4 °C prior to staining.

#### Induction of antigen specific CD8 + T cell lines from healthy donor PBMCs

Immature dendritic cells (DCs) were generated from healthy donor PBMCs by overnight incubation with GM-CSF and IL-4. Mature DCs were generated by overnight incubation of immature DCs with TNF-α, IL-1ß, IL-6, and prostaglandin E-2. Mature DCs were loaded with 1 µg/mL of pooled HLA-restricted peptides and incubated for 4 h at 37 °C in a MACSmix™ Tube Rotator (Miltenyi Biotec). CD8^+^ T cells were isolated from autologous PBMCs using the EasySep™ Human CD8^+^ T Cell Enrichment Kit (STEMCELL Technologies). Following incubation, peptide-loaded DCs were irradiated at 4000 RAD. Lines were generated (10/donor) by combining irradiated DCs with CD8^+^ T cells and IL-21. Lines were incubated at 37 °C and maintained every 2-3 days with CTL, IL-2, IL-7, and IL-15. 10-14 days following line generation, stimulation 2 was carried out by combining irradiated PBMCs from the same donors with cells from stimulation 1, pooled peptides at 1ug/mL, and IL-21. This process was repeated for a total of three stimulations. In the REP, CD8 + T cells were expanded in the presence of anti-CD3 Abs (100 ng/mL), anti-CD28 Abs (100 ng/mL), IL2 (20 IU/mL), IL7 (10 ng/mL), IL15 (10 ng/mL), irradiated mixed PBMCs from the three healthy donors and TM-LCL lines. The cell culture medium was half-replenished every 3 days up to 14 days of culture.

#### UV-exchange & ELISA assay

4 μl of refolded MHCs loaded with photo-cleavable peptides (MHC-J) (0.5 μM) were mixed with 1 μl of the target peptide (50 μM) to be exchanged with, and exposed to 365 nm UV for 60 min on ice. An ELISA assay was performed to quantify the UV-exchange efficiency as described below. Briefly, NeutraAvidin plates (ThermoFisher, 15507) were washed four times with wash buffer (phosphate buffered saline (PBS) containing 0.05% Tween‐20 and 0.1% BSA) and blocked with blocking buffer (PBS containing 2% BSA) for 1 h at 37˚C. Wells were incubated with either 100 μL UV-exchanged samples or MHC-J at 5 nM for 1 h at 37˚C. A folded MHC complex made in house at eight serial two‐fold dilutions, starting from 32 nM were used as positive controls. After washing four times with wash buffer, wells were incubated with HRP‐conjugated β2m antibodies (Rockland, 1:5k dilution) in blocking buffer for 1 h at 37˚C. Wells were washed four times again before incubating with 100 μL TMB substrate (Seracare, 5120‐0047). The TMB reaction was quenched after 5 min using 1 M sulfuric acid. The OD at 450 nm was measured on a Spectramax Plate Reader. UV exchange efficiency was calculated as background-subtracted OD450 of UV-exchanged samples divided by background-subtracted OD450 of the MHC-J sample.

#### Tetramer pool-based sorting and in vitro T-cell expansion

The monomer SCTs were individually tetramerized with PE or APC labeled streptavidin at a 4:1 molar ratio for 30 min at 4 C. Biotin was added at an 8:1 molar ratio to streptavidin to block unoccupied biotin binding sites on streptavidin. Each SCT tetramer was pooled at an individual tetramer concentration of 50 nM. The PBMCs were freshly thawed, washed, and stained with calcein violet AM (100 nM) and CD8-FITC antibody (1 μg/ml) for 10 min at 4 °C followed by incubation with a pool of SCT tetramers (each, 20 nM). Antigen-specific CD8^+^ T cells labeled with calcein violet AM, anti-CD8 antibody, and SCT-tetramer-PE were bulk sorted into the tube containing a cell culture medium (FACSAria Fusion). The sorted CD8^+^ T cells were then expanded using the REP. The cell culture medium was half-replenished every 3 days up to 14 days of culture. The tetramer-sorted and expanded T cells were stained with individual tetramer, and tetramer-positive CD8^+^ T cell populations were analyzed by flow cytometry (Attune NxT). For IFN-γ assay after expansion, cells were overnight recovered and incubated with 1 µg/mL peptide for 12 h at 37 °C. Cells were stained for tetramer and CD8 expression, followed by intracellular staining for IFN-γ production.

#### Production of DNA-barcoded Dextramer and TCR sequencing

DNA-barcoded dextramer was produced by mixing the DNA-barcoded and PE-labeled klickmer (Immudex) with the SCT monomer at a 1:28 molar ratio for 30 min at 4 °C, followed by adding excess biotins. The CD8^+^ T cells were isolated by MACS and stained with cell hashtag antibodies (BioLegend). The hashtagged and pooled T cells were stained with CD8-FITC antibody (1 μg/ml) and a dextramer pool. CD8 and PE positive T cell populations were sorted and loaded onto a Chromium Next GEM chip G (10× Genomics, 1000120). Chromium Single Cell Kits (10× Genomics, 1000165) were utilized to analyze the hashtag, TCR, and antigen sequences simultaneously from the same cell. Full-length cDNA, along with cell barcode identifiers, were PCR-amplified, and sequencing libraries were prepared and normalized. The constructed library was sequenced on the Novaseq platform (Illumina).

#### Cloning TCR constructs and transduction

The TCR α and β DNA constructs were PCR amplified, and Gibson assembled and ligated into the pRRL-SIN Lentiviral vector. The sequence-verified plasmid DNA is transfected into 293 T cells line along with packaging plasmids to produce lentiviral particles. CD8^+^ T cells were isolated from healthy PBMCs using the EasySep™ Human CD8^+^ T Cell Enrichment Kit (STEMCELL Technologies) and activated with Dynabeads™ CD3/CD28 (Life Technologies) at a ratio of 1:1 of bead to cell for 48 h in the presence of IL2 (100 IU/mL).

To generate C4ba TCR, cells were spinfected (2500 RPM, 30 °C, 90 min) with lentiviral particles encoding the C4ba TCR in the presence of IL-2 and polybrene (Sigma-Aldrich). Cells were maintained every 2-3 days with CTL media and IL-2. Following CD8^+^, tetramer-positive sort, and REP, cells were stained with tetramer, CD8, and propidium iodide.

To generate the SARS-CoV2-specific T cell lines, a complex of Cas9 and sgRNA targeting the first exon of the TRAC (AGAGTCTCTCAGCTGGTACA) and TRBC locus (GCTGTCAAGTCCAGTTCTAC) was prepared and electroporated into CD8^+^ T cells at 3 pulses of 1600 V and 10 ms using Neon electroporator (Thermo Fisher Scientific). Cells were then transduced with lentiviral particles encoding the SARS-CoV2 TCR, and the medium was exchanged after 12 h. Cells were maintained every 2–3 days with CTL media and IL-2. The transduced T-cells were stained with tetramer and CD8 and FACS-sorted and expanded using a REP.

#### Peptide synthesis and purification

All peptides were synthesized by using standard Fmoc solid-phase peptide synthesis procedures using Wang resin. A Titan 357 (Aapptec) instrument was used to couple all Fmoc-protected standard amino acids. After synthesis was complete, peptides were cleaved by mixing with 10 mL cleave solution (95% Trifluoroacetic acid +2.5% Triethylsilane +2.5% DI with vigorous stirring for 2 h. The resulting solution was added to 40 mL of diethyl ether (Acros Organics, 615080-0040), and the product was then pelleted by centrifugation, dried in the air, and then resuspended in a 30% acetonitrile (Fisher, A955-4). Peptides were purified on a Waters Autopurification system, which isolate compounds based on MS peaks corresponding to protonated [M + 1H]^+^ and [M + 2H]^2+^. The resulting peptides were lyophilized and resuspended at a concentration of up to 10 mM peptide in DMSO.

#### Cytokines and LDH measurement

Cloned 100 k CD8^+^ T cells were co-cultured with HLA-matched 50 k T2 cells line pulsed with 1 μM of the peptide in 200 µL of CTL media. After 16 h of incubation, each 50 µL of the supernatant was extracted for analysis by standard ELISA protocols for TNF-α (R&D Systems, DY210-05), IFN-γ (R&DSystems, DY285B-05), and Granzyme B (R&D Systems, DY2906-05) and by Non-Radioactive Cytotoxicity Assay (Promega, G1780) for LDH measurement. Every experiment was duplicated.

#### IncuCyte cell killing assay

Prior to the coculture of target cells and effector cells, dead cell removal was applied to both target and effector cells to deplete any apoptotic cells. Target cells were stained with Cytolight Red dye (Sartorius, 4705) at a concentration of 0.33 µM in the presence of 1 µM of the peptide. A hundred microlitres of CTL media containing CD28 antibody (100 ng/mL) and Caspase-3/7 dye (5 µM, Sartorius, 4440) was added into the well of a 96-well plate. 50 k of peptide-pulsed T2 target cells and unlabeled 100 k of CD8^+^ T-cells were resuspended in each 50 µL of CTL media and were added into the well. In order to extract the dead cell signal from only APCs, total overlap (green and red) and red object areas (square micrometers per well) were quantified, and the ratio of overlap to red signal was interpreted as the killing of the target cells. Cells were imaged at four positions per well every 20 min for 12 h. Killing curves for TCR clonotypes were plotted over time and fitted by asymmetric sigmoidal nonlinear regression.

#### Statistics and reproducibility

All data were presented as the mean ± SD of three experimental replicates (unless otherwise indicated within figure legends) and analyzed using Student’s unpaired two-tailed *t*-test. A *p* value of <0.05 denoted statistically significant differences. The sample size used to derive each statistic was provided in the figure legends.

### Reporting summary

Further information on research design is available in the Nature Portfolio Reporting Summary linked to this article.

## Supplementary information

Heath_Peer Review File

Supplementary Information

Description of Additional Supplementary Files

Supplementary Data

Reporting Summary

## Data Availability

The data generated in this study are provided in the main text, Supplementary Information, Supplementary Data Excel file, or available from the authors upon request. Raw images of the SDS-PAGE results from Figs. 1c and 2b may be found in Supplementary Figs. 1e and 2c, respectively. The processed sequencing data generated in this study have been deposited in the ArrayExpress database at EMBL-EBI (www.ebi.ac.uk/arrayexpress) under accession number E-MTAB-11229.

## Code availability

The custom Python scripts used for data analysis are publicly available on GitHub.
