# Supplementary material for: Large libraries of single-chain trimer peptide-MHCs enable antigen-specific CD8+ T cell discovery and analysis
Source: Commun Biol. 2023 May 16;6:528. doi: 10.1038/s42003-023-04899-8 (PMC10186326; doi:10.1038/s42003-023-04899-8)
Supplement: Supplementary file 2 — Supplementary Information [file 42003_2023_4899_MOESM2_ESM.pdf]

Large libraries of single-chain trimer peptide-MHCs enable antigen-specific CD8+ T cell discovery and analysis

Authors: William Chour[1,2†], Jongchan Choi[1†], Jingyi Xie[1,3], Mary E. Chaffee[4], Thomas M. Schmitt[4], Kathryn Finton[4], Diana C. DeLucia[5], Alexander M. Xu[1,6], Yapeng Su[1,6], Daniel G. Chen[1,7], Rongyu Zhang[1,8], Dan Yuan[1,8], Sunga Hong[1], Alphonsus H.C. Ng[1,6], Jonah Z. Butler[4], Rick A. Edmark[1], Lesley C. Jones[1], Kim M. Murray[1], Songming Peng[9], Guideng Li[10,11,12], Roland K. Strong[4], John K. Lee[5,13], Jason D. Goldman[14,15], Philip D. Greenberg[4,10,16], and James R. Heath[1,8*].

SUPPLEMENTAL FIGURES and TABLES

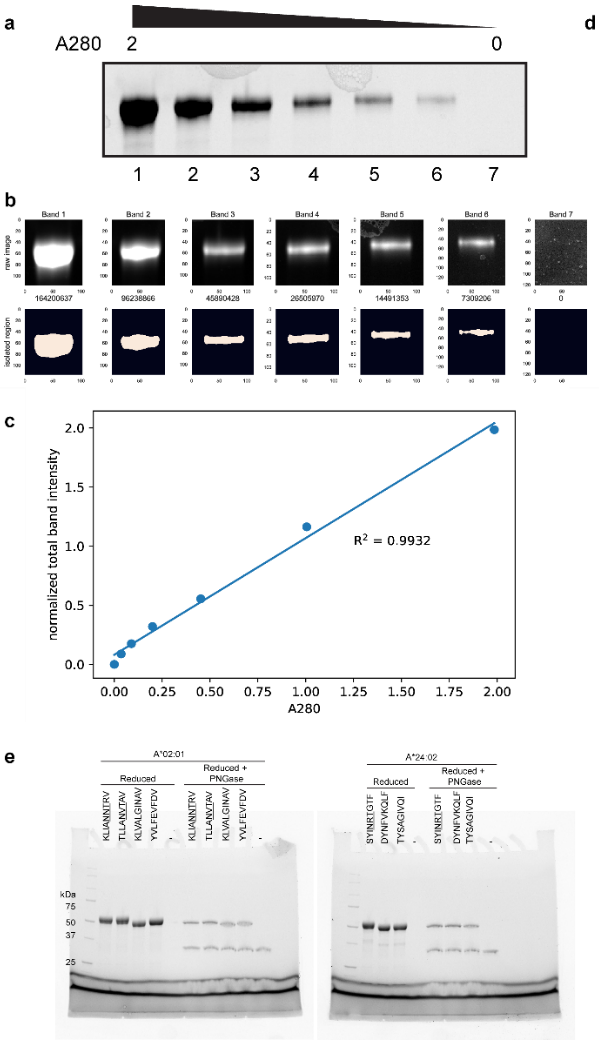

d

ID	peptide	netMHC affinity (nM)	D3 SCT	D8 SCT	UV pMHC
01	RLFPYALHK	6.11	[green]	[green]	[orange]
02	RTREFTAKK	48.98			[orange]
03	TLWEQFLEK	65.93			[orange]
04	CRLFPYALHK	12.56			
05	ALLPPPPLAK	10.37	[green]	[green]	[orange]
06	KIYTGEKPYK	7.13	[green]	[green]	[orange]
07	ILFRTPSVAK	7	[green]	[green]	[orange]
08	KMFCAGYPK	16.23	[green]	[green]	[orange]
09	RLFSALNSHK	7.44	[green]	[green]	[orange]
10	RVDFSATWYK	13.16			[orange]
11	AVRVLFPGIK	226.32			
12	ATYKGVPYEVK	51.99		[green]	[orange]
13	KVYDIAFSR	67.93	[green]	[green]	[orange]
14	KEAADPLAFK	58.02			
15	GIKAHPVLK	56.37			
16	GLSFHLQSSK	16.57	[green]	[green]	[orange]
17	GVYTKEFLY	170.48			
18	KAPVGHFYK	243.75		[green]	[orange]
19	KINRALTAAK	27.19	[green]	[green]	[orange]
20	KIVEMSTSK	74.54		[green]	[orange]
21	KLHVASLSFR	47.04	[green]	[green]	[orange]
22	ATRYNYTSEK	18.27			[orange]
23	SLSNFPQLK	21.62			
24	RKSNRVKTCK	230.96			
25	SLKTNQTLQLK	746.81			
26	SLHAHGLSYK	8.22	[green]		[orange]
27	RMFLRFQKK	10.2	[green]	[green]	[orange]
28	LLPPPPLAK	52.31		[green]	
29	RVLFPGIK	405.56			
30	RVWDVSGLRK	26.52	[green]	[green]	[orange]
31	RTREFTAKKIK	573.89			
32	AINRPTVLK	15.78			
33	LLFKAGEMRK	11.78	[green]	[green]	[orange]
34	ASFFGVHSK	34.98			
35	HLFDIQGLPK	15.85	[green]	[green]	[orange]
36	KLRDEISLAK	20.79		[green]	[orange]
37	ATNYIYVLNK	17.32			

[green] high SCT expression
[orange] sufficient UV exchange (stable product)

Supplementary Figure 1. SCT yield quantification and a comparison of D3 and D8 SCTs and UV-exchanged pMHCs. **a**. SDS-PAGE image of WT1 SCTs, titrated across a range of approximately 2.0 to 0 absorbance units (280 nm). **b**. Manually cropped areas of each band from reference gel (top). Isolated regions of bands identified by Python script (bottom). Integers below the top row represent the relative calculated intensity of each identified band (bottom). **c**. Scatterplot showing correlation of A280 values and calculated band intensity from SDS-PAGE for each reference band. **d**. A comparison of D3 and D8 SCTs and UV-exchanged pMHCs on the thirty-seven A*03:01-restricted private neoantigens predicted for a melanoma patient. Relative expression of D3 and D8 SCTs were quantified according to methods outlined in (a-c, see Methods) and then converted into a binary format (based upon yield being above intensity threshold of 0.15) to identify stable/high-yield pMHCs (columns 4 & 5).Stabilization of the UV-exchanged pMHCs for the same peptides was measured via an ELISA assay, also converted into a binary format (ELISA efficiency > 30%) (column 6). **e**. Raw images of reduced SDS-PAGE results from **Fig. 1c**.

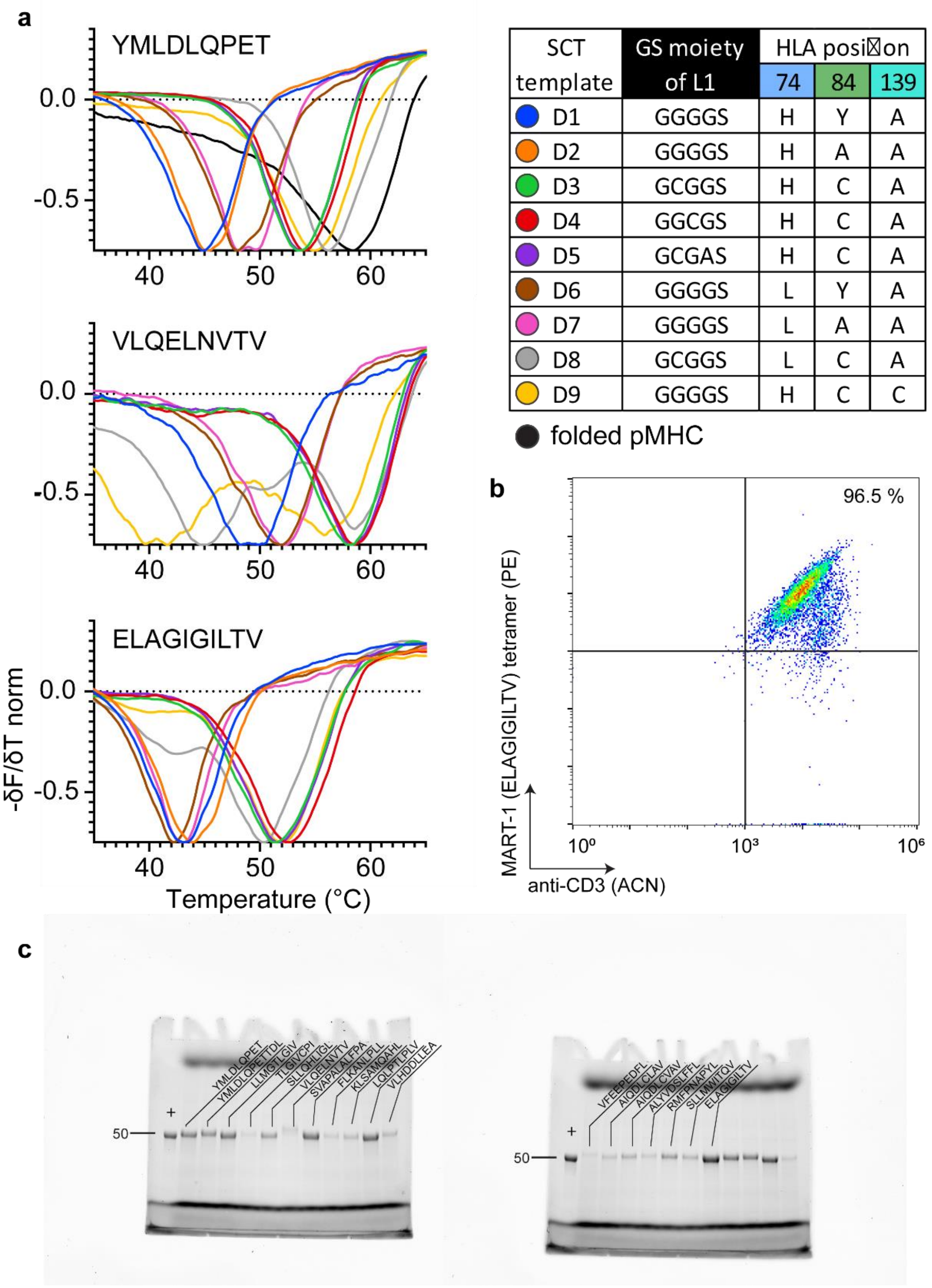

SCT template	GS moiety of L1	HLA position		
		74	84	139
D1	GGGGS	H	Y	A
D2	GGGGS	H	A	A
D3	GCGGS	H	C	A
D4	GGCGS	H	C	A
D5	GCGAS	H	C	A
D6	GGGGS	L	Y	A
D7	GGGGS	L	A	A
D8	GCGGS	L	C	A
D9	GGGGS	H	C	C

Supplementary Figure 2. Characterization of SCT thermal stability and functional evaluation of SCT tetramer. **a.** Thermal melting curves of SCTs. The negative of the change in fluorescence over the change in temperature (−δF/δT, first derivative) is measured for all SCT templates made against three peptides of **Fig. 2b** that expressed at a sufficient yield for purification. Melting curves are representative of a biological triplicate per SCT. **b**. Capturing of MART1-specific F5 TCR-transduced Jurkat cells with the MART1 epitope presenting SCT tetramers (D3 template). **c.** Raw images of reduced SDS-PAGE results from **Fig. 2b**.

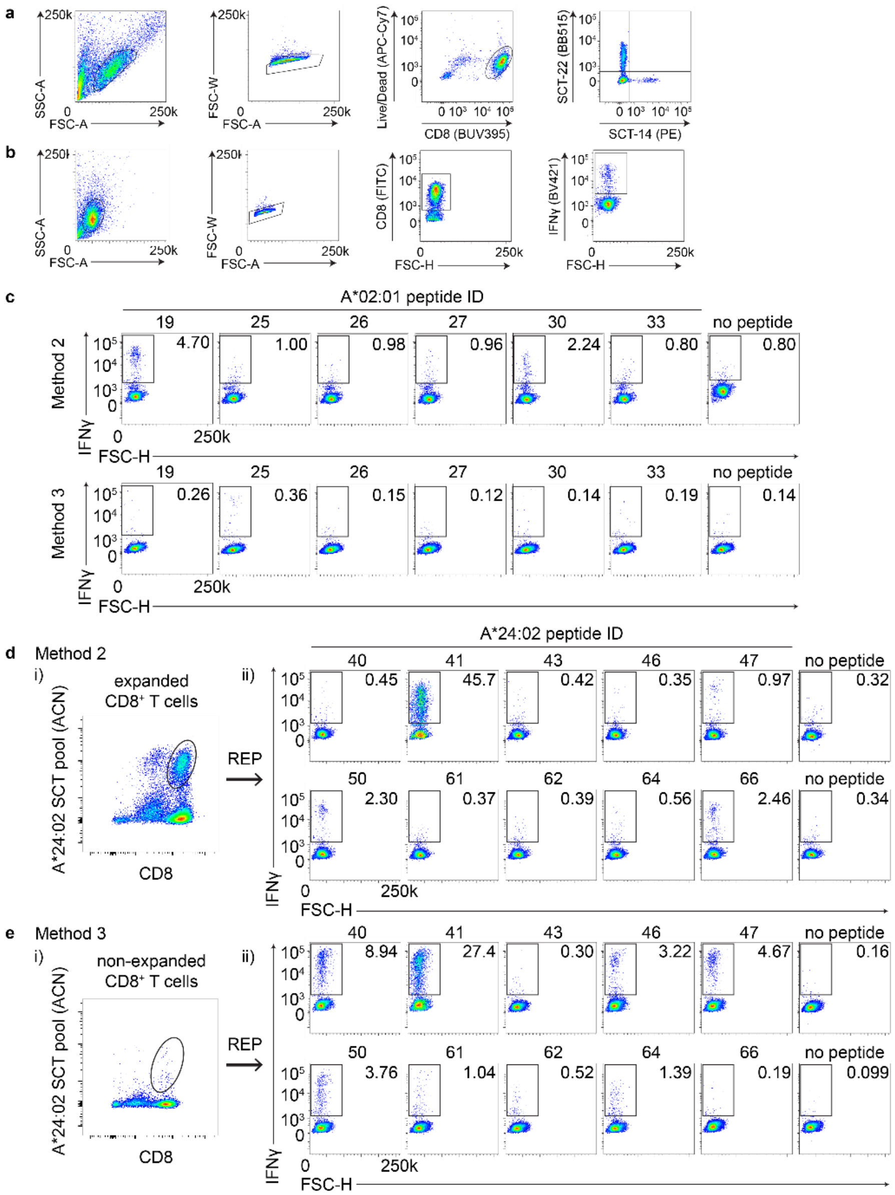

Supplementary Figure 3. Flow cytometric analysis of antigen specific CD8⁺ T cell response against commonly reported viral epitopes. **a**. Gating strategies for flow cytometry plots of CD8+ T cells captured by 5-color pooled SCT tetramers from peptide-stimulated and expanded CD8+ T cells. Lymphocytes, single cells, CD8+ T cells and tetramer positive populations were sequentially gated. **b**. Gating strategies for flow cytometry plots of IFNγ+ CD8+ cells after peptide stimulation. **c**. Representative flow cytometry plots of IFNγ+ A*02:01-restricted CD8+ T cells after peptide stimulation. These cells were initially obtained by single-color pooled SCT tetramer binding and REP expansion (see **Fig. 3 c.i andd.i**). **d,e**. Representative flow cytometry plots of A*24:02-restricted CD8+ T cells initially captured by single-color pooled SCT tetramers from peptide-stimulated and expanded CD8+ T cells (**d.i**) (Method #2) or from non-stimulated, non-expanded CD8+ T cells (**e.i**) (Method #3). Subsequent flow plots represent IFNγ+ cells after peptide stimulation of expanded cells from the previously captured tetramer-positive subset (**d.ii, e.ii**). Peptide sequences found in **Supplementary Table 2**. Values in flow cytometry plots indicate the percentage of total cell population captured within outlined box. FSC, forward scatter; SSC, side scatter; A, area; W, width; H, height; SCT; single chain trimer; IFNγ, interferon γ; REP, rapid expansion protocol.

a

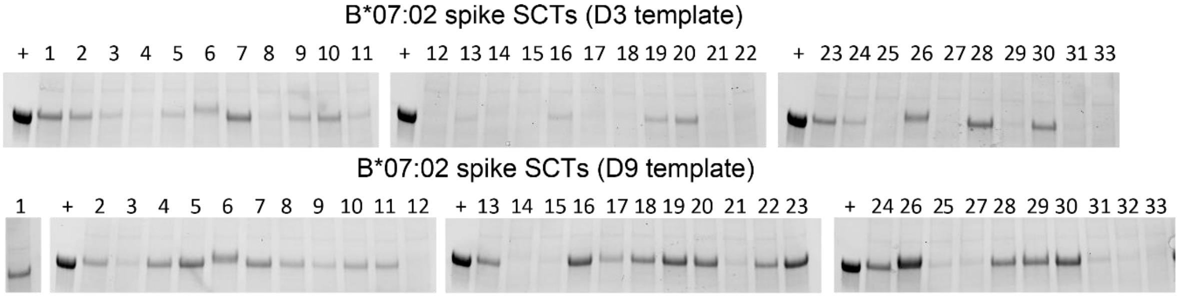

b

ID	peptide	D3	D9
1	SPRRARSVA		
2	SPRRARSVAS		
3	NSPRRARSVA		
4	MIAQYTSAL		
5	YPDKVFRSSV		
6	IPTNFTISV		
7	FPQSAPHGVV		
8	LPVSMTKTSV		
9	TNSPRRARSVA		
10	SPRRARSVASQ		
11	RARSVASQSI		
12	QPYRVVVLSF		
13	LPPAYTNSF		
14	FPNITNLCPF		
15	WPWYIWLGF		
16	TPINLVRDL		
17	VPVAIHADQL		
18	TPCSFGGVSV		
19	YPDKVFRSSVL		
20	KPFERDISTEI		
21	RAAEIRASA		
22	SIIAYTMSL		
23	LPQGFSALEPL		
24	MIAQYTSALL		
25	NSPRRARSVAS		
26	AIPTNFTISV		
27	SEPVLKGVKL		
28	APGQTGKIA		
29	FPQSAPHGV		
30	FPQSAPHGVVF		
31	ITRFQTLLAL		
32	VVNQNAQAL		
33	SLSSTASAL		

c

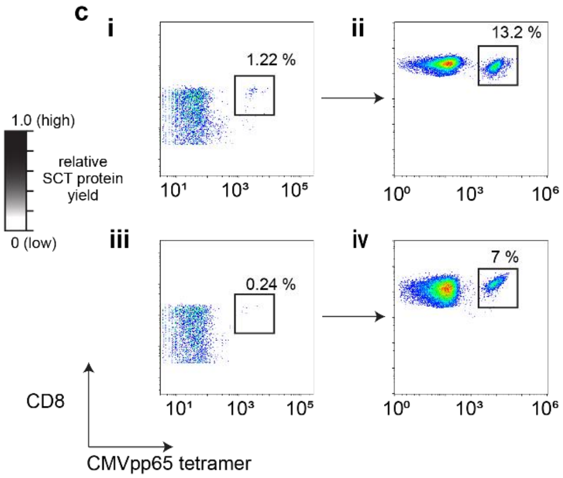

Supplementary Figure 4. Expression and characterization of B*07:02 spike SCTs and their usage to capture and expand antigen-specific CD8⁺ T cell populations. a. Reduced SDS-PAGE of B*07:02 spike epitope SCTs using two templates (B_Y84C corresponds to template D3 and A_Y84C_A139C corresponds to template D9 (DS-SCT) of table in Figure 2.B). +, purified WT1 SCT. **b**. SCT yield heatmap comparing relative expression results for the two templates. **c**. Flow cytometry plots of CMVpp65 specific CD8⁺ T cell populations before (i and iii) and after (ii and iv) tetramer-guided sorting and expansion in the two A*02:01 COVID-19 patients. The inset box in each plot shows the frequency of CMVpp65 specific CD8⁺ T cell population.

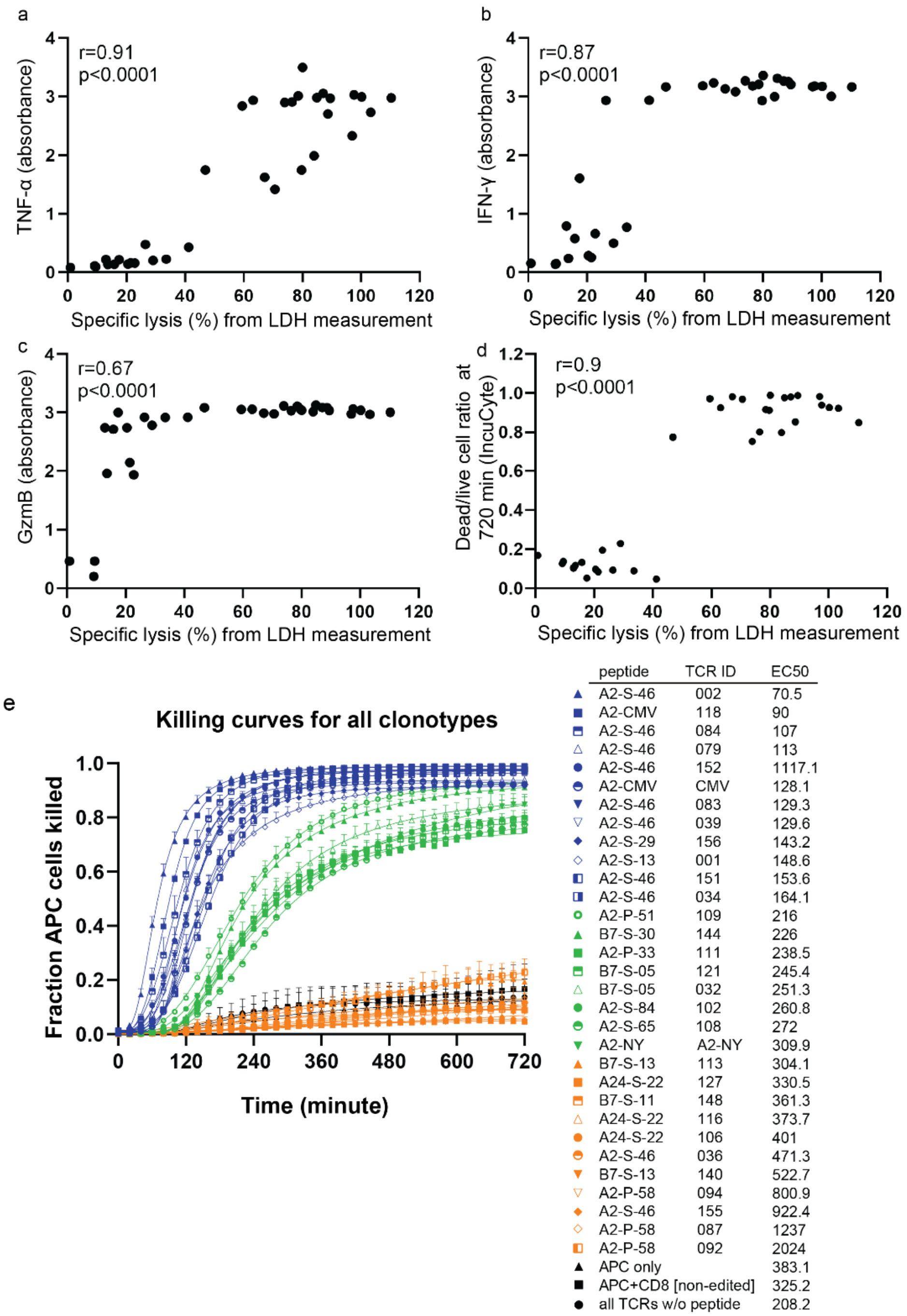

Supplementary Figure 5. Linear regression analysis of T cell functional data and curve fitting for time course killing curves. a-c. Scatter plots showing positive correlations between all three functional cytokines (TNFα, IFNγ, and GzmB) and the assayed LDH levels. **d**. A scatter plot of data obtained from the two cytotoxic assays (incuCyte killing assay and non-radioactive cytotoxicity assay for LDH measurement). **e**. Nonlinear regression curve fitting and analysis of EC50 from IncuCyte kinetic curves. Error bars represent standard deviation from independent triplicate measurements. The list of peptide, internal TCR ID, and calculated EC50 values are in table. LDH, lactate dehydrogenase; TNFα, tumor necrosis factor α; IFNγ, interferon γ; GzmB, granzyme B; NY, SLLMWITQC (NY-ESO-1); CMV, NLVPMVATV; A2, A*02:01; B7, B*07:02; APC, antigen-presenting cell; P, PLpro; S, spike.

Supplementary Table 1. Tumor-associated peptides and sources.

peptide ID	peptide	protein	PubMed ref ID
1	YMLDLQPET	E7 (PPV-9)	29669936
2	YMLDLQPETTDL	E7 (PPV-9)	27693214
3	LLMGTLGIV	E7 (PPV-9)	26418056
4	TLGIVCPI	E7 (PPV-9)	8617954
5	SLLQHLIGL	MART	22424782
6	VLQELNVTV	Myeloblastin	PMC1534319
7	SVAPALALFPA	LB-ADIR-1F	17234742
8	FLKANLPLL	MTG8b	11122105
9	KLSAMQAHL	Foxp3	23812418
10	LQLPTLPLV	Foxp3	19456276
11	VLHDDLLEA	HA-1/A2	18698046
12	VFEEPEDFL	Foxp3	23812418
13	AIQDLCLAV	nucleophosmin	31291378
14	AIQDLCVAV	nucleophosmin	31291378
15	ALYVDSLFFL	PRAME	PMC2195886
16	RMFPNAPYL	WT1	PMC5722674
17	SLLMWITQV	NY-ESO-1	PMC5941317
18	ELAGIGILTV	MART-1	16860654

Supplementary Table 2. Viral/bacterial A*02:01 and A*24:02 peptides used for SCT production

ID	peptide	antigen	HLA	yield
1	LLFGYPVYV	HTLV-1 Tax	A*02:01	0.35
2	KLVALGINAV	HCV	A*02:01	0.50
3	GLCTLVAML	EBV-BLMF1	A*02:01	0.00
4	WLSLLVPFV	HBV-SAg	A*02:01	0.03
5	YVLDHLIVV	EBV-BRLF1	A*02:01	0.45
6	SITEVECFL	Human polyomavirus 2 protein	A*02:01	0.29
7	FLLSLGIHL	HBV	A*02:01	0.36
8	GILGFVFTL	Flu-M1	A*02:01	0.05
9	SLFNTVATL	HIV gag	A*02:01	0.52
10	YLLFEVFDV	AdV 11 Hexon	A*02:01	0.45
11	LLFEVFDVV	AdV 11 Hexon	A*02:01	0.79
12	YVLFEVFDV	AdV 11 Hexon	A*02:01	0.43
13	FLDKGTYTL	EBV BALF4	A*02:01	0.12
14	YLQQNWWTL	EBV-LMP1-2	A*02:01	0.48
15	YLLEMLWRL	EBV-LMP1-1	A*02:01	0.12
16	FLYALALLL	EBV-LMP1-2	A*02:01	0.10
17	VLEETSVML	CMV-IE1	A*02:01	0.00
18	TLNAWVKVV	HIV-gag	A*02:01	0.08
19	AIMDKNIIL	Influenza NS1	A*02:01	0.33
20	KLIANNTRV	M. tuberculosis Ag85A	A*02:01	0.38
21	ALWALPHAA	varicella-zoster IE62 593-601	A*02:01	0.05
22	NLVPMVATV	CMV-pp65	A*02:01	0.70
23	FMYSDFHFI	Influenza A	A*02:01	0.56
24	YLLPGWKL	Rota-VP3	A*02:01	0.00
25	NMLSTVLGV	Flu-PB1	A*02:01	0.61
26	SLMDPAILTSL	Rota-VP1	A*02:01	1.42
27	TLLANVTAV	Rota-VP6	A*02:01	0.85
28	FMDILTTCVET	CMV-IE1-2	A*02:01	0.23
29	QMWQARLTV	CMV-pp65-2	A*02:01	0.07
30	SLISGMWLL	Rota-VP2-1	A*02:01	0.26
31	LLNYILKSV	Rota-VP7-1	A*02:01	0.14
32	LMNGQQIFL	CMV-pp65-3	A*02:01	0.26
33	FLDSEPHLL	Rota-NSP1	A*02:01	0.15
34	ALWGPDPAAA	Proinsulin precursor 15-24	A*02:01	0.23
35	TLDYKPLSV	EBV BMRF1	A*02:01	0.07
36	CLGGLLTMV	EBV-LMP2A	A*02:01	0.04
37	TYFNLGNKF	AdV 11 Hexon (37-45)	A*24:02	0.80
38	VYSGSIPYL	AdV 11 Hexon (696-704)	A*24:02	0.18

39	TYFSLNNKF	AdV 5 Hexon (37-45)	A*24:02	0.93
40	DYNFVKQLF	EBV BMLF1 (320-328)	A*24:02	0.84
41	TYPVLEEMF	EBV BRLF1 (198-206)	A*24:02	1.39
42	RYSIFFDYM	EBV EBNA3A (246-254)	A*24:02	0.05
43	TYSAGIVQI	EBV EBNA3B (217-225)	A*24:02	0.84
44	IYVLVMLVL	EBV LMP2 (222-230)	A*24:02	0.00
45	PYLFWLAAI	EBV LMP2 (131-139)	A*24:02	0.00
46	TYGPVFMSL	EBV LMP2 (419-427)	A*24:02	1.13
47	TYGPVFMCL	EBV LMP2 (419-427)	A*24:02	0.64
48	EYLVSFGVW	HBV core (117-125)	A*24:02	0.59
49	KYTSFPWLL	HBV pol (756-764)	A*24:02	0.23
50	QYDPVAALF	HCMV pp65 (341-349)	A*24:02	0.51
51	EYVLLLFLL	HCV E2 (717-725)	A*24:02	0.00
52	PFHCSFHTI	HHV-6B U54 (267-275)	A*24:02	0.00
53	RYLRDQQLL	HIV env gp160 (584-592)	A*24:02	0.19
54	RYLKDQQLL	HIV env (67-75)	A*24:02	0.20
55	RYPLTFGW	HIV nef (134-141)	A*24:02	0.26
56	VYDFAFRDL	HPV16 E6 (49-57)	A*24:02	0.13
57	FFQFCPLIF	HTLV-1 Env (43788)	A*24:02	0.63
58	LFGYPVYVF	HTLV-1 Tax (43819)	A*24:02	0.51
59	PYKRIEELL	HTLV-1 Tax (187-195)	A*24:02	0.00
60	SFHSLHLLF	HTLV-1 Tax (301-309)	A*24:02	0.83
61	YYLEKANKI	Influenza PA (130-138)	A*24:02	0.99
62	SYLIRALTL	Influenza PB1 (216-224)	A*24:02	0.39
63	RYTKTTYWW	Influenza PB1 (430-438)	A*24:02	0.11
64	SYINRTGTF	Influenza PB1 (482-490)	A*24:02	1.02
65	RYGFVANF	Influenza PB1 (498-505)	A*24:02	0.00
66	TYQWIIRNW	Influenza PB2 (549-557)	A*24:02	0.90

Supplementary Table 3. A*02:01 spike peptides used for SCT production

ID	peptide	domain	span	length	predicted nM affinity	yield	ID
01	YLQPRTFLL	NTD	269-277	9	5.4	0.33	01
02	FQFCNDPFL	NTD	133-141	9	9.2	1.19	02
03	FIAGLIAIV	TM	1220-1228	9	10.3	0.09	03
04	FVSNGTHWFV	CD	1095-1104	10	13.1	0.53	04
05	SIIAYTMSL	S2	691-699	9	13.5	0.88	05
06	KLNDLCFTNV	RBD	386-395	10	15.3	0.21	06
07	RLQSLQTYV	CH	1000-1008	9	16.7	0.18	07
08	YHLMSFPQSA	S2	1047-1056	10	17.9	0.08	08
09	YIKWPWYIWL	S2/TM	1209-1218	10	18.8	0.00	09
10	YIWLGFIAGL	TM	1215-1224	10	20.5	0.09	10
11	FELLHAPATV	RBD/S1	515-524	10	21	0.16	11
12	FTISVTTEI	S2	718-726	9	25.4	1.74	12
13	LLFNKVTLA	FP	821-829	9	25.4	1.74	13
14	HLMSFPQSA	S2	1048-1056	9	26.4	1.24	14
15	FVFLVLLPLV	S	2-11	10	32.6	0.00	15
16	VLNDILSRL	HR1	976-984	9	33.6	0.11	16
17	FIEDLLFNKV	FP	817-826	10	36	0.11	17
18	GYLQPRTFLL	NTD	268-277	10	36.1	0.13	18
19	KIADYNYKL	RBD	417-425	9	36.1	0.50	19
20	VVFLHVTYV	S2	1060-1068	9	36.6	0.08	20
21	RLDKVEAEV	HR1/CH	983-991	9	39	0.23	21
22	FVFLVLLPL	S	2-10	9	41.7	0.00	22
23	GFIAGLIAIV	TM	1219-1228	10	56.9	0.15	23
24	YQDVNCTEV	S1	612-620	9	57.8	0.83	24
25	FLHVTYVPA	S2	1062-1070	9	65.2	0.73	25
26	MIAQYTSAL	S2	869-877	9	69	0.92	26
27	KLPDDFTGCV	RBD	424-433	10	77.1	0.26	27
28	GLIAIVMVTI	TM	1223-1232	10	80.8	0.00	28
29	KQIYKTPPI	S2	786-794	9	85.6	0.61	29
30	EFQFCNDPFL	NTD	132-141	10	87.8	0.00	30
31	TKLNDLCFTNV	RBD	385-395	11	91.2	0.00	31
32	SVTTEILPV	S2	721-729	9	113.1	0.90	32
33	YLQPRTFLLK	NTD	269-278	10	118.8	0.54	33
34	MQMAYRFNGI	S2/HR1	900-909	10	120.9	0.39	34
35	FQFCNDPFLGV	NTD	133-143	11	126.8	1.09	35
36	GRLQSLQTYV	CH	999-1008	10	128.4	0.13	36
37	GLTVLPPLL	S2	857-865	9	130.4	0.13	37
38	FIAGLIAIVMV	TM	1220-1230	11	136.3	0.09	38

39	VFVSNGTHWFV	CD	1094-1104	11	137.7	0.00		39
40	WYIWLGFIAGL	TM	1214-1224	11	140.1	0.00		40
41	SFELLHAPATV	RBD/S1	514-524	11	140.8	0.10		41
42	KQLSSNFGA	HR1	964-972	9	141.2	0.22		42
43	FIAGLIAIVM	TM	1220-1229	10	143	0.10		43
44	TLDSKTQSL	NTD	109-117	9	153.6	0.12		44
45	MFVFLVLLPL	S	1-10	10	157.8	0.00		45
46	RLITGRLQSL	CH	995-1004	10	167	1.06		46
47	GYHLMSFPQSA	S2	1046-1056	11	172.6	0.09		47
48	NLNESLIDL	HR2	1192-1200	9	177.3	0.24		48
49	VLYENQKLI	HR1	915-923	9	194.4	0.16		49
50	KLIANQFNSA	HR1	921-930	10	195.6	1.09		50
51	VTWFHAIHV	NTD	62-70	9	202	0.17		51
52	DLLFNKVTLA	FP	820-829	10	202	0.08		52
53	QSIIAYTMSL	S2	690-699	10	207.4	0.00		53
54	SRLDKVEAEV	HR1/CH	982-991	10	221.2	0.08		54
55	QYIKWPWYIWL	S2/TM	1208-1218	11	223.4	0.00		55
56	TLLALHRSYL	NTD	240-249	10	227.8	0.33		56
57	MFVFLVLLPLV	S	1-11	11	229	0.00		57
58	GLIAIVMVT	TM	1223-1231	9	242	0.06		58
59	YTNSFTRGV	NTD	28-36	9	245.1	0.18		59
60	ITSGWTFGA	S2	882-890	9	265.1	0.11		60
61	KNLNESLIDL	HR2	1191-1200	10	268.8	0.07		61
62	GVVFLHVTYV	S2	1059-1068	10	272.4	0.12		62
63	LLIVNNATNV	NTD	117-126	10	272.8	0.16		63
64	SLSSTASAL	HR1	937-945	9	273.3	0.22		64
65	KIYSKHTPI	NTD	202-210	9	288.5	0.36		65
66	FVSGNCDVV	CD	1121-1129	9	290.4	0.12		66
67	FQFCNDPFLG	NTD	133-142	10	296.2	1.17		67
68	KLNDLCFTNVY	RBD	386-396	11	302.6	0.16		68
69	VLSFELLHA	RBD	512-520	9	302.6	0.11		69
70	AIPTNFTISV	S2	713-722	10	324.5	0.11		70
71	VLLPLVSSQCV	S/NTD	16-Jun	11	325.3	0.49		71
72	LLALHRSYL	NTD	241-249	9	329.1	0.23		72
73	IITTDNTFV	CD	1114-1122	9	338.8	0.10		73
74	IMLCCMTSC	TM/CT	1232-1240	9	349	0.13		74
75	VFLHVTYVPA	S2	1061-1070	10	377.3	0.00		75
76	ELLHAPATV	RBD/S1	516-524	9	380.7	0.99		76
77	VLHSTQDLFL	NTD	47-56	10	382.2	0.00		77
78	VLYNSASFST	RBD	367-376	10	388.9	0.29		78
79	YRVVVLSFEL	RBD	508-517	10	391.5	0.00		79
80	SFIEDLLFNKV	FP	816-826	11	394.6	0.05		80
81	FLPFFSNVT	NTD	55-63	9	398.9	0.15		81

82	SVLNDILSRL	HR1	975-984	10	399.3	0.22		82
83	ALEPLVDLPI	NTD	222-231	10	405.6	0.07		83
84	ALQIPFAMQM	S2	893-902	10	415.7	1.76		84
85	RVVVLSFEL	RBD	509-517	9	419.8	0.12		85
86	FTISVTTEIL	S2	718-727	10	419.8	1.66		86
87	FELLHAPATVC	RBD/S1	515-525	11	435.6	0.09		87
88	YIWLGFIAGLI	TM	1215-1225	11	436.3	0.00		88
89	KTSVDCTMYI	S2	733-742	10	438.1	0.00		89
90	GIYQTSNFRV	S1	311-320	10	449	0.09		90
91	MIAQYTSALL	S2	869-878	10	453	1.10		91
92	LGFIAGLIAIV	TM	1218-1228	11	457.3	0.08		92
93	VGYLQPRTFLL	NTD	267-277	11	461.4	0.06		93
94	WTAGAAAYYV	NTD	258-267	10	463.3	0.08		94
95	SWMESEFRV	NTD	151-159	9	475.3	0.09		95
96	NFTISVTTEI	S2	717-726	10	501.6	0.13		96

Supplementary Table 4. A*24:02 spike peptides used for SCT production

ID	peptide	domain	span	length	predicted nM affinity	yield		ID
01	KWPWYIWLGF	TM	1211-1220	10	9	0.16		01
02	LYNSASFSTF	RBD	368-377	10	12.3	0.31		02
03	QYIKWPWYI	TM	1208-1216	9	13.2	0.32		03
04	QYIKWPWYIW	TM	1208-1217	10	14.9	0.00		04
05	VYSTGSNVF	S1	635-643	9	19	0.46		05
06	VYSSANNCTF	NTD	159-168	10	21.9	0.55		06
07	CYFPLQSYGF	RBD	488-497	10	26.6	0.00		07
08	NYNYLYRLF	RBD	448-456	9	28.9	0.49		08
09	RVYSTGSNVF	S1	634-643	10	36.3	0.16		09
10	IKWPWYIWLGF	TM	1210-1220	11	36.3	0.00		10
11	PFAMQMAYRF	S2	897-906	10	38.9	0.00		11
12	YFPLQSYGF	RBD	489-497	9	41.5	0.00		12
13	KWPWYIWLGFI	TM	1211-1221	11	42.1	0.78		13
14	EQYIKWPWYI	TM	1207-1216	10	45.3	0.33		14
15	YEQYIKWPWYI	TM	1206-1216	11	55.8	0.31		15
16	VLYNSASFSTF	RBD	367-377	11	64.3	0.00		16
17	YYVGYLQPRTF	NTD	265-275	11	73.3	0.26		17
18	LYNSASFSTFK	RBD	368-378	11	76.3	0.73		18
19	VYSTGSNVFQ	S1	635-644	10	93.3	0.21		19
20	EQYIKWPWYIW	TM	1207-1217	11	96.2	0.38		20
21	KYEQYIKWPW	TM	1205-1214	10	110.2	0.53		21
22	FAMQMAYRF	S2	898-906	9	111.5	0.54		22
23	QYIKWPWYIWL	TM	1208-1218	11	115.5	0.40		23
24	IYQTSNFRV	S1	312-320	9	124.6	0.00		24
25	TYVPAQEKNF	S2	1066-1075	10	137.3	0.68		25
26	GYLQPRTFLL	NTD	268-277	10	138.1	0.35		26
27	RVYSSANNCTF	NTD	158-168	11	143.7	0.17		27
28	RFPNITNLCPF	RBD	328-338	11	149.3	0.13		28
29	KWPWYIWLG	TM	1211-1219	9	152	0.00		29

30	PYRVVVLSF	RBD	507-515	9	152.4	0.00	30
31	VYAWNRKRI	RBD	350-358	9	160.3	0.00	31
32	VYSSANNCTFE	NTD	159-169	11	172.5	0.31	32
33	GNYNYLYRLF	RBD	447-456	10	174.6	0.22	33
34	YYHKNNKSW	NTD	144-152	9	223.5	0.32	34
35	NYNYLYRLFR	RBD	448-457	10	233.2	0.20	35
36	IYSKHTPINL	NTD	203-212	10	233.7	0.00	36
37	IWLGFIAGLI	TM	1216-1225	10	239	0.00	37
38	NCYFPLQSYGF	RBD	487-497	11	243.2	0.16	38
39	IYKTPPIKDF	S2	788-797	10	252.1	0.24	39
40	YNSASFSTF	RBD	369-377	9	252.4	0.61	40
41	WRVYSTGSNVF	S1	633-643	11	274.1	0.00	41
42	CYFPLQSYGFQ	RBD	488-498	11	299.8	0.53	42
43	TFEYVSQPF	NTD	167-175	9	337.1	0.58	43
44	YFPLQSYGFQ	RBD	489-498	10	349.3	0.78	44
45	RVYSTGSNVFQ	S1	634-644	11	371.1	0.32	45
46	CFTNVYADSF	RBD	391-400	10	371.3	0.43	46
47	EYVSQPFLM	NTD	169-177	9	373.2	0.53	47
48	VFVSNGTHWF	CD	1094-1103	10	377.5	0.45	48
49	YLQPRTFLL	NTD	269-277	9	406.7	0.25	49
50	IPFAMQMAYRF	S2	896-906	11	427.8	0.30	50
51	VYYHKNNKSW	NTD	143-152	10	488.9	0.00	51

Supplementary Table 5. B*07:02 spike peptides used for SCT production

ID	peptide	domain	span	length	predicted nM affinity	yield	ID
01	SPRRARSVA	S1/S2	680-688	9	4.2	0.25	01
02	SPRRARSVAS	S1/S2	680-689	10	18.4	0.27	02
03	NSPRRARSVA	S1/S2	679-688	10	20.1	0.09	03
04	MIAQYTSAL	S2	869-877	9	28.9	0.36	04
05	YPDKVFRSSV	NTD	38-47	10	50.2	0.60	05
06	IPTNFTISV	S2	714-722	9	95.1	0.47	06
07	FPQSAPHGVV	S2	1052-1061	10	99.6	0.44	07
08	LPVSMTKTSV	S2	727-736	10	100	0.22	08
09	TNSPRRARSVA	S1/S2	678-688	11	104.1	0.12	09
10	SPRRARSVASQ	S1/S2	680-690	11	136.2	0.23	10
11	RARSVASQSI	S1/S2	683-692	10	147	0.21	11
12	QPYRVVVLSF	RBD	506-515	10	163.1	0.00	12
13	LPPAYTNSF	NTD	24-32	9	168	0.32	13
14	FPNITNLCPF	RBD	329-338	10	175.2	0.00	14
15	WPWYIWLGF	TM	1212-1220	9	193.4	0.00	15
16	TPINLVRDL	NTD	208-216	9	202.5	0.59	16
17	VPVAIHADQL	S1	620-629	10	210.8	0.19	17
18	TPCSFGGVSV	S1	588-597	10	249.9	0.35	18
19	YPDKVFRSSVL	NTD	38-48	11	255.4	0.59	19
20	KPFERDISTEI	RBD	462-472	11	286	0.50	20
21	RAAEIRASA	CH	1014-1022	9	317.7	0.08	21
22	SIIAYTMSL	S2	691-699	9	328	0.29	22
23	LPQGFSALEPL	NTD	216-226	11	337.2	0.68	23
24	MIAQYTSALL	S2	869-878	10	350.9	0.41	24
25	NSPRRARSVAS	S1/S2	679-689	11	361.2	0.08	25
26	AIPTNFTISV	S2	713-722	10	381	0.88	26
27	SEPVLKGVKL	CP	1261-1270	10	388.2	0.08	27
28	APGQTGKIA	RBD	411-419	9	407.8	0.38	28
29	FPQSAPHGV	S2	1052-1060	9	409.9	0.45	29
30	FPQSAPHGVVF	S2	1052-1062	11	413.7	0.62	30
31	ITRFQTLLAL	NTD	235-244	10	444.5	0.10	31
32	VVNQNAQAL	HR1	951-959	9	472.6	0.05	32
33	SLSSTASAL	HR1	937-945	9	492.4	0.00	33

Supplementary Table 6. A*02:01 PLpro peptides used for SCT production

ID	peptide	span	length	predicted nM affinity	yield	ID
01	ILFTRFFYV	1514-1522	9	3.2	0.23	01
02	YILFTRFFYV	1513-1522	10	3.8	0.21	02
03	YIFFASFYYV	1566-1575	10	5	0.13	03
04	YLATALLTL	857-865	9	5.1	0.17	04
05	WLMWLIINL	1545-1553	9	6.6	0.00	05
06	YMPYFFTLL	1350-1358	9	7.1	0.21	06
07	FLTENLLLYI	430-439	10	7.2	0.92	07
08	KLINIIIWFL	1407-1416	10	7.3	0.00	08
09	QLFFSYFAV	1530-1538	9	10	0.09	09
10	LLSAGIFGA	330-338	9	10.1	0.48	10
11	AYILFTRFFYV	1512-1522	11	10.5	0.17	11
12	MQLFFSYFAV	1529-1538	10	10.7	0.32	12
13	WLMWLIINLV	1545-1554	10	11	0.11	13
14	YLKLTDNVYI	213-222	10	12.5	0.24	14
15	LMWLIINLV	1546-1554	9	13.3	0.00	15
16	YMPYFFTLLL	1350-1359	10	13.9	0.10	16
17	FLGRYMSAL	824-832	9	14.9	0.32	17
18	KLMPVCVET	569-577	9	15.1	0.90	18
19	FVMMSAPPA	986-994	9	15.1	1.32	19
20	YVWKSYVHV	1574-1582	9	15.2	0.85	20
21	ILFTRFFYVL	1514-1523	10	16	0.10	21
22	QLEMELTPVV	193-202	10	17	0.22	22
23	YLNSTNVTI	1452-1460	9	17.2	0.10	23
24	IIWFLLLSV	1412-1420	9	19	0.00	24
25	QLMCQPILL	1745-1753	9	19	0.41	25
26	YLVQQESPFV	978-987	10	19.3	1.31	26
27	QMAPISAMV	1555-1563	9	19.6	0.00	27
28	CYLATALLTL	856-865	10	19.8	0.08	28
29	KLLHKPIVWHV	1166-1176	11	20.5	0.79	29
30	YLFDESGEFKL	88-98	11	22.2	0.74	30
31	YILFTRFFYVL	1513-1523	11	22.3	0.11	31
32	MYIFFASFYYV	1565-1575	11	22.7	0.09	32
33	MQLFFSYFA	1529-1537	9	23.1	0.47	33
34	ILGTVSWNL	549-557	9	23.6	0.00	34
35	SWLMWLIINL	1544-1553	10	24.9	0.00	35
36	LLHKPIVWHV	1167-1176	10	27	0.26	36
37	ILLLDQALV	1751-1759	9	27.1	0.87	37
38	FISNSWLMWL	1540-1549	10	29	0.00	38

39	KMFDAYVNT	1771-1779	9	30.2	0.88		39
40	SLIYSTAAL	1424-1432	9	30.8	0.93		40
41	YVWKSYVHVV	1574-1583	10	32.5	0.55		41
42	FELDERIDKV	25-34	10	34.8	0.09		42
43	KFLTENLLLYI	429-439	11	36.4	0.22		43
44	SKLINIIIWFL	1406-1416	11	38.4	0.09		44
45	VLLAPLLSA	325-333	9	40.8	0.93		45
46	SQLMCQPILL	1744-1753	10	42.1	0.34		46
47	FLKKDAPYI	460-468	9	42.2	0.18		47
48	TLVTMPLGYV	626-635	10	43.5	0.75		48
49	NTFSSTFNV	1778-1786	9	43.6	0.28		49
50	YSQLMCQPILL	1743-1753	11	45.4	0.12		50
51	TLATHGLAAV	1303-1312	10	47.6	0.34		51
52	NYMPYFFTLL	1349-1358	10	48.7	0.12		52
53	AMVRMYIFFA	1561-1570	10	49	0.10		53
54	IMQLFFSYFA	1528-1537	10	51.3	0.10		54
55	KLVSSFLEM	367-375	9	52.5	0.40		55
56	SLDNVLSTFI	1807-1816	10	52.8	0.29		56
57	YTVELGTEV	42-50	9	53.7	0.64		57
58	MLAKALRKV	494-502	9	54.2	0.50		58
59	VLSTFISAA	1811-1819	9	55.3	0.13		59
60	YLATALLTLQ	857-866	10	56.4	0.18		60
61	FITESKPSV	396-404	9	57.5	0.48		61
62	LVAEWFLAYI	1505-1514	10	59.3	0.13		62
63	IIIWFLLLSV	1411-1420	10	59.9	0.00		63
64	SWLMWLIINLV	1544-1554	11	61.5	0.08		64
65	VQMAPISAMV	1554-1563	10	61.8	0.32		65
66	IMQLFFSYFAV	1528-1538	11	67.1	0.07		66
67	QLMCQPILLL	1745-1754	10	67.6	0.43		67
68	FGLVAEWFLA	1503-1512	10	67.7	0.49		68
69	YLDGADVTKI	780-789	10	67.8	0.21		69
70	NLAKHCLHV	291-299	9	68	0.14		70
71	YIFFASFYYVW	1566-1576	11	68.1	0.09		71
72	ILAYCNKTV	896-904	9	68.7	0.17		72
73	LLLSVCLGSL	1416-1425	10	71.4	0.36		73
74	LLDQALVSDV	1753-1762	10	80.1	0.27		74
75	FLKKDAPYIV	460-469	10	80.8	0.21		75
76	LLAPLLSAGI	326-335	10	80.9	0.58		76
77	KLINIIIWFLL	1407-1417	11	81	0.17		77
78	SLKVPATVSV	650-659	10	85.2	0.11		78
79	KLDGVVCTEI	1065-1074	10	87.1	0.18		79
80	IFFASFYYV	1567-1575	9	87.8	0.07		80
81	YLAVFDKNL	356-364	9	89	0.42		81

82	YMRSLKVPATV	647-657	11	89.2	0.13	82
83	AVIKTLQPV	60-68	9	89.6	0.61	83
84	ALLTLQQIEL	861-870	10	91.7	1.06	84
85	FCLEASFNYL	1391-1400	10	93.3	0.49	85
86	TLNDLNETL	619-627	9	95.6	0.14	86
87	ALQDAYYRA	876-884	9	100.3	0.08	87
88	NYMPYFFTLLL	1349-1359	11	101.3	0.10	88
89	FLTENLLLYID	430-440	11	102	1.00	89
90	GLVAEWFLA	1504-1512	9	105.3	0.24	90
91	YVNTFSSTFNV	1776-1786	11	110.1	0.46	91
92	VLGLAAIMQL	1522-1531	10	111.3	0.09	92
93	PLLSAGIFGA	329-338	10	111.6	0.13	93
94	YVDNSSLTI	1281-1289	9	115.3	0.41	94
95	RKLMPVCVET	568-577	10	121.6	0.08	95
96	WLDDDSQQTV	161-170	10	124.2	0.30	96
97	ILFTRFFYVLG	1514-1525	11	125.3	0.14	97
98	LINIIIWFL	1408-1417	9	126.6	0.08	98
99	NLACEDLKPV	1220-1230	10	128.4	0.22	99
100	KLSHQSDIEV	1838-1848	10	128.5	0.40	100
101	LMWLIINLVQ	1546-1556	10	131.1	0.13	101
102	YYVWKSYVHV	1573-1583	10	131.2	0.15	102
103	SLINTLNDL	615-624	9	133	1.49	103
104	GYLKLTDNVYI	212-223	11	135.4	0.10	104
105	NLAKHCLHVV	291-301	10	137.4	0.19	105
106	TLNDLNETLV	619-629	10	139.8	0.22	106
107	FTRFFYVLGL	1516-1526	10	142.2	0.28	107
108	NSWLMWLIINL	1543-1554	11	149.1	0.00	108
109	WLMWLIINLVQ	1545-1556	11	154.2	0.09	109
110	QLFFSYFAVH	1530-1540	10	156.8	0.00	110
111	FLNKVVSTT	1327-1336	9	157	0.32	111
112	GYLNSTNVTI	1451-1461	10	164.2	0.09	112
113	VLTAVVIPT	477-486	9	168.2	0.16	113
114	LLLQLCTFT	1357-1366	9	169.2	0.32	114
115	PQLEMELTPVV	192-203	11	170.5	0.21	115
116	VLPNDDTLRV	802-812	10	172.5	0.08	116
117	NCYLATALLTL	855-866	11	173.3	0.13	117
118	SAFYILPSI	533-542	9	179.7	0.13	118
119	TLYCIDGALL	1026-1036	10	180.9	0.10	119
120	LMCQPILLL	1746-1755	9	184.2	0.35	120
121	IIWFLLLSVC	1412-1422	10	188	0.00	121
122	FSYFAVHFI	1533-1542	9	195.1	0.13	122

123	SFLGRYMSAL	823-833	10	196.9	0.07		123
124	MPLGYVTHGL	630-640	10	197.4	0.10		124
125	LILAYCNKTV	895-905	10	199.2	0.15		125
126	MQLFFSYFAVH	1529-1540	11	214.2	0.12		126
127	QLEMELTPV	193-202	9	215.1	0.23		127
128	EILGTVSWNL	548-558	10	217.3	0.13		128
129	PILLLDQALV	1750-1760	10	222.8	0.17		129
130	FLAYILFTRF	1510-1520	10	226.9	0.00		130
131	KYLVQQESPFV	977-988	11	230.2	0.09		131
132	LLTLQQIEL	862-871	9	239.6	0.77		132
133	FISNSWLMWLI	1540-1551	11	240.2	0.00		133
134	TTIQTIVEV	182-191	9	241.3	0.69		134
135	KIAEIPKEEV	384-394	10	247.7	0.19		135
136	YLKSPNFSKL	1399-1409	10	248.8	0.07		136
137	KLMPVCVETKA	569-580	11	249.5	0.82		137
138	YIATNGPLKV	272-282	10	250.5	0.09		138
139	YMRSLKVPA	647-656	9	251.4	0.10		139
140	MELTPVVQTI	196-206	10	255.7	0.11		140
141	TLYCIDGAL	1026-1035	9	267.7	0.08		141
142	IDLDEWSMAT	77-87	10	281.2	0.09		142
143	YLNSTNVTIA	1452-1462	10	289.4	0.09		143
144	MQVESDDYI	265-274	9	294	0.24		144
145	EVLLAPLLSA	324-334	10	296.2	0.07		145
146	SLETIQITI	1485-1494	9	297.5	0.25		146
147	GLVAEWFLAYI	1504-1515	11	300.4	0.20		147
148	FLLLSVCLGS	1415-1425	10	300.8	0.00		148
149	YYVWKSYVHVV	1573-1584	11	302.2	0.00		149
150	LLSVCLGSL	1417-1426	9	305.3	0.11		150
151	VNTFSSTFNV	1777-1787	10	308.6	0.09		151
152	YMPYFFTLLLQ	1350-1361	11	309	0.09		152
153	TLQPVSELL	64-73	9	315	0.32		153
154	FLLLSVCLGSL	1415-1426	11	324.7	0.06		154
155	NLYDKLVSSFL	363-374	11	325.4	0.28		155
156	LLYIDINGNL	436-446	10	327.3	0.28		156
157	FGDDTVIEV	7-16	9	328.5	0.30		157
158	RTIKVFTTV	748-757	9	330.4	0.75		158
159	LIYSTAALGV	1425-1435	10	330.8	0.14		159
160	FLEMKSEKQV	372-382	10	334.8	0.09		160
161	LLSAGIFGAD	330-340	10	351.2	0.32		161
162	GSLIYSTAAL	1423-1433	10	358.6	0.94		162
163	YLFDESGEF	88-97	9	360	0.68		163

164	PFVMMSAPPA	985-995	10	363.4	0.20		164
165	TFELDERIDKV	24-35	11	368.3	0.08		165
166	CLGSLIYST	1421-1430	9	375.2	0.07		166
167	KLINIIIWF	1407-1416	9	382.9	0.10		167
168	WLIINLVQMA	1548-1558	10	395.1	0.00		168
169	LVQMAPISAMV	1553-1564	11	402.1	0.49		169
170	MVRMYIFFA	1562-1571	9	404	0.00		170
171	KLMPVCVETK	569-579	10	404.3	0.94		171
172	NLHPDSATL	444-453	9	411.9	0.12		172
173	HFISNSWLMWL	1539-1550	11	415.1	0.00		173
174	VLMSNLGMPS	1434-1444	10	417.1	0.45		174
175	LVQQESPFV	979-988	9	419.2	0.19		175
176	KLTCATTRQV	1923-1933	10	430.8	0.08		176
177	FVCDNIKFA	1112-1121	9	432.8	0.63		177
178	DKLVSSFLEM	366-376	10	447	0.00		178
179	TIVEVQPQL	186-195	9	447.8	0.58		179
180	KSVNITFEL	19-28	9	453.2	0.82		180
181	VLNEKCSAYTV	34-45	11	457.4	0.10		181
182	FLAYILFTR	1510-1519	9	460.6	0.05		182
183	TNYMPYFFTLL	1348-1359	11	467.8	0.00		183
184	NIIIWFLLLSV	1410-1421	11	470.4	0.00		184
185	TMSYLFQHA	913-922	9	481.5	0.00		185
186	FPDLNGDVVAI	1142-1153	11	489.5	0.10		186
187	LLLDQALVSDV	1752-1763	11	491.8	1.24		187
188	YPQVNGLTSI	840-850	10	491.9	0.00		188
189	KLASHMYCS	97-106	9	493.4	0.38		189
190	HNLAKHCLHV	290-300	10	494.6	0.00		190
191	YLVQQESPFVM	978-989	11	497.2	1.48		191

Supplementary Table 7. List of TCRs, cognate antigens, and their groupings

TCR ID	EC50	IncuCyte	Peptide	Peptide	TCR group	HLA	Protein	TRAV	CDR3a	TRAJ	TRBV	CDR3b	TRBJ	TRBC	Patient
002	70.5	0.987808	A2-S-46	RLITGRLQSL	F/F	A*02:01	S	TRAV39	CAVDANNDMRF	TRAJ43	TRBV12-4	CASSPSYNEQFF	TRBJ2-1	TRBC2	GB18622 (healthy donor)
118	90.0	0.971802	A2-CMV	NLVPMVATV	F/F	A*02:01	S	TRAV24	CACPPGDYKLSF	TRAJ20	TRBV7-8	CASSLDIPSYNEQFF	TRBJ2-1	TRBC2	InCoV005-CV
084	107.0	0.988771	A2-S-46	RLITGRLQSL	F/F	A*02:01	S	TRAV22	CAAQSNMEYGNKLVF	TRAJ47	TRBV5-4	CASSSPDRGGTNEKLFF	TRBJ1-4	TRBC1	GB17457 (healthy donor)
079	113.0	0.938558	A2-S-46	RLITGRLQSL	F/F	A*02:01	S	TRAV19	CALSEGYNFNKFYF	TRAJ21	TRBV20-1	CSGEGGDTEAFF	TRBJ1-1	TRBC1	InCoV005-CV
152	117.1	0.982336	A2-S-46	RLITGRLQSL	F/F	A*02:01	S	TRAV17	CATDLKTSYDKVIF	TRAJ50	TRBV20-1	CSARDPGLEQNIQYF	TRBJ2-4	TRBC2	InCoV005-CV
A2-CMV	128.1	0.921789	A2-CMV	NLVPMVATV	F/F	A*02:01	CMV								InCoV194-T2
083	129.3	0.980294	A2-S-46	RLITGRLQSL	F/F	A*02:01	S	TRAV20	CAVQAAREYNFNKFYF	TRAJ21	TRBV3-1	CASSQEGDRVTEAFF	TRBJ1-1	TRBC1	GB18622 (healthy donor)
039	129.6	0.981115	A2-S-46	RLITGRLQSL	F/F	A*02:01	S	TRAV17	CATDLKTSYDKVIF	TRAJ50	TRBV20-1	CSARSPGELNIQYF	TRBJ2-4	TRBC2	InCoV002-CV
156	143.2	0.915092	A2-S-29	KQIYKTPPI	F/F	A*02:01	S	TRAV4	CLVPSEQAGTALIF	TRAJ15	TRBV20-1	CSASSTHSNQPQHF	TRBJ1-5	TRBC1	InCoV002-CV
001	148.6	0.925161	A2-S-13	LLFNKVTLA	F/F	A*02:01	S	TRAV9-2	CALNDYKLSF	TRAJ20	TRBV28	CASSLTGGGYEQYF	TRBJ2-7	TRBC2	GB18622 (healthy donor)
151	153.6	0.968874	A2-S-46	RLITGRLQSL	F/F	A*02:01	S	TRAV25	CAGHPKTSYDKVIF	TRAJ50	TRBV20-1	CSARDPGLEQNIQYF	TRBJ2-4	TRBC2	InCoV005-CV
034	164.1	0.976687	A2-S-46	RLITGRLQSL	F/F	A*02:01	S	TRAV10	CVVSPINYGQNFVF	TRAJ26	TRBV3-1	CASSQEQEPHQETQYF	TRBJ2-5	TRBC2	InCoV002-CV
109	216.0	0.924808	A2-P-51	TLATHGLAAV	M/F	A*02:01	P	TRAV8-4	CAVLVPGNQFYF	TRAJ49	TRBV19	CASSIAGNNEQFF	TRBJ2-1	TRBC2	InCoV002-CV
144	226.0	0.911642	B7-S-30	FPQSAPHGVVF	M/F	B*07:02	S	TRAV17	CATEDNAGNMLTF	TRAJ39	TRBV9	CASSVGGGGSTDTQYF	TRBJ2-3	TRBC2	InCoV006-CV
111	238.5	0.752108	A2-P-33	MQLFFSYFA	M/F	A*02:01	P	AV38-2/D	CAYGDDKIIF	TRAJ30	TRBV19	CASSIALSTFQPQHF	TRBJ1-5	TRBC1	InCoV002-CV
121	245.4	0.797145	B7-S-05	YPDKVFRSSV	M/F	B*07:02	S	TRAV27	CAGGYNNNDMRF	TRAJ43	TRBV6-6	CASSLGEPQHF	TRBJ1-5	TRBC1	InCoV006-CV
032	251.3	0.85282	B7-S-05	YPDKVFRSSV	M/F	B*07:02	S	TRAV27	CAGGYNNNDMRF	TRAJ43	TRBV9	CASSVGGGGSTDTQYF	TRBJ2-3	TRBC2	InCoV006-CV
102	260.8	0.800694	A2-S-84	ALQIPFAMQM	M/F	A*02:01	S	TRAV5	CAETLNTGFQKLVF	TRAJ8	TRBV28	CASRTLRGHVSEAFF	TRBJ1-1	TRBC1	InCoV005-CV
108	272.0	0.774104	A2-S-65	KIYSKHTPI	M/F	A*02:01	S	TRAV12-2	CAVDDFSGGTSYGKLTF	TRAJ52	TRBV28	CASSFPGYGYTF	TRBJ1-2	TRBC1	InCoV002-CV
A2-NY	309.9	0.848193	A2-NY	SLLMWITQC	M/F	A*02:01	NY-ESO-1	TRAV21-1	CAVRPLYGGSYIPTF	TRAJ6-1	TRBV6-5	CASSYVGNTGELF	TRBJ2-2	TRBC2	healthy donor
113	304.1	0.194018	B7-S-13	LPPAYTNSF	NF	B*07:02	S	TRAV8-4	CAVSFFNQAGTALIF	TRAJ15	TRBV12-4	CASSKKGLSNEQFF	TRBJ2-1	TRBC2	InCoV006-CV
127	330.5	0.046486	A24-S-22	FAMQMAYRF	NF	A*24:02	S	TRAV24	CARNSGGYQKVTF	TRAJ13	TRBV5-6	CASSLMVNEQYF	TRBJ2-7	TRBC2	InCoV001-CV
148	361.3	0.088618	B7-S-11	RARSVASQSI	NF	B*07:02	S	TRAV8-2	CVVRDGGYNKLIF	TRAJ4	TRBV10-3	CATFTGNTEAFF	TRBJ1-1	TRBC1	InCoV006-CV
116	373.7	0.092381	A24-S-22	FAMQMAYRF	NF	A*24:02	S	TRAV24	CAFCGGTSYGKLTF	TRAJ52	TRBV5-6	CASSLDGTGREKLFF	TRBJ1-4	TRBC1	InCoV009-CV
106	401.0	0.051551	A24-S-22	FAMQMAYRF	NF	A*24:02	S	TRAV24	CAFSGGSNYKLTF	TRAJ53	TRBV5-6	CASSFGTPSTDTQYF	TRBJ2-3	TRBC2	InCoV009-CV
036	471.3	0.116178	A2-S-46	RLITGRLQSL	NF	A*02:01	S	TRAV12-2	CAGRPYDKIIF	TRAJ30	TRBV3-1	CASSQEQEPHQETQYF	TRBJ2-5	TRBC2	InCoV002-CV
140	522.7	0.096399	B7-S-13	LPPAYTNSF	NF	B*07:02	S	TRAV16	CALKTIKAAGNKLTF	TRAJ17	TRBV5-5	CASSSLDGRLGYTF	TRBJ1-2	TRBC1	InCoV006-CV
094	800.9	0.103591	A2-P-58	MLAKALRKV	NF	A*02:01	P	TRAV35	CAGPIGTSYDKVIF	TRAJ50	TRBV12-4	CASSSAHYGYTF	TRBJ1-2	TRBC1	InCoV002-CV
155	922.4	0.083552	A2-S-46	RLITGRLQSL	NF	A*02:01	S	TRAV35	CAGQLVASYDKVIF	TRAJ50	TRBV20-1	CSARTGTSEYNEQFF	TRBJ2-1	TRBC2	InCoV005-CV
087	1237.0	0.131551	A2-P-58	MLAKALRKV	NF	A*02:01	P	RAV29/DV	CAASDDNYGQNFVF	TRAJ26	TRBV7-9	CASSPDDRESSYNEQFF	TRBJ2-1	TRBC2	InCoV002-CV
092	2024.0	0.228311	A2-P-58	MLAKALRKV	NF	A*02:01	P	TRAV35	CAGLNQGAQKLVF	TRAJ54	TRBV7-9	CASSPDDRESSYNEQFF	TRBJ2-1	TRBC2	InCoV002-CV

*F/F: fast and functional, M/F: medium and functional, N/F: non-functional
