# Supplementary material for: Large libraries of single-chain trimer peptide-MHCs enable antigen-specific CD8+ T cell discovery and analysis
Source: Commun Biol. 2023 May 16;6:528. doi: 10.1038/s42003-023-04899-8 (PMC10186326; doi:10.1038/s42003-023-04899-8)
Supplement: Supplementary file 3 — Description of Additional Supplementary Files [file 42003_2023_4899_MOESM3_ESM.pdf]

Description of Additional Supplementary Files

File name: Supplementary Data

Description: Source data behind numerical plots in the paper.
