# Supplementary material for: Large libraries of single-chain trimer peptide-MHCs enable antigen-specific CD8+ T cell discovery and analysis
Source: Commun Biol. 2023 May 16;6:528. doi: 10.1038/s42003-023-04899-8 (PMC10186326; doi:10.1038/s42003-023-04899-8)
Supplement: Supplementary file 5 — Reporting Summary [file 42003_2023_4899_MOESM5_ESM.pdf]

nature portfolio

Corresponding author(s): James R. Heath

Last updated by author(s): Apr 28, 2023

Reporting Summary

Nature Portfolio wishes to improve the reproducibility of the work that we publish. This form provides structure for consistency and transparency in reporting. For further information on Nature Portfolio policies, see our Editorial Policies and the Editorial Policy Checklist.

Statistics

For all statistical analyses, confirm that the following items are present in the figure legend, table legend, main text, or Methods section.

n/a	Confirmed	
☐	☒	The exact sample size (*n*) for each experimental group/condition, given as a discrete number and unit of measurement
☐	☒	A statement on whether measurements were taken from distinct samples or whether the same sample was measured repeatedly
☒	☐	The statistical test(s) used AND whether they are one- or two-sided<br>*Only common tests should be described solely by name; describe more complex techniques in the Methods section.*
☒	☐	A description of all covariates tested
☐	☒	A description of any assumptions or corrections, such as tests of normality and adjustment for multiple comparisons
☐	☒	A full description of the statistical parameters including central tendency (e.g. means) or other basic estimates (e.g. regression coefficient) AND variation (e.g. standard deviation) or associated estimates of uncertainty (e.g. confidence intervals)
☒	☐	For null hypothesis testing, the test statistic (e.g. *F*, *t*, *r*) with confidence intervals, effect sizes, degrees of freedom and *P* value noted<br>*Give P values as exact values whenever suitable.*
☒	☐	For Bayesian analysis, information on the choice of priors and Markov chain Monte Carlo settings
☒	☐	For hierarchical and complex designs, identification of the appropriate level for tests and full reporting of outcomes
☒	☐	Estimates of effect sizes (e.g. Cohen's *d*, Pearson's *r*), indicating how they were calculated

Our web collection on statistics for biologists contains articles on many of the points above.

Software and code

Policy information about availability of computer code

Data collection	Single cell sequencing data was obtained using a NovaSeq S4.
Data analysis	Droplet-based sequencing data for T-cell receptor sequences were aligned and quantified using the Cell Ranger Single-Cell Software Suite (10x Genomics) against the GRCh38 human VDJ reference genome. Prism 8 (GraphPad) was used for the analysis of the data. Custom Python scripts were used to generate primer sequences for SCT plasmid production and to analyze gel electrophoresis band intensities of the expressed SCT proteins. These scripts can be found online at https://github.com/wchour/nat-comms-bio-SCT.

For manuscripts utilizing custom algorithms or software that are central to the research but not yet described in published literature, software must be made available to editors and reviewers. We strongly encourage code deposition in a community repository (e.g. GitHub). See the Nature Portfolio guidelines for submitting code & software for further information.

Data

Policy information about availability of data

All manuscripts must include a data availability statement. This statement should provide the following information, where applicable:

- Accession codes, unique identifiers, or web links for publicly available datasets
- A description of any restrictions on data availability
- For clinical datasets or third party data, please ensure that the statement adheres to our policy

All data are available in the main text or in the supplementary materials. TCR sequencing data have been deposited in the ArrayExpress database at EMBL-EBI (www.ebi.ac.uk/arrayexpress) under accession number E-MTAB-11229. All other information is available from the corresponding authors upon reasonable request.

Human research participants

Policy information about studies involving human research participants and Sex and Gender in Research.

Reporting on sex and gender	The sex was reported by the participant and recorded by the physician.<br><br>Samples were selected considering only HLA-haplotyping result that matched with our study, not by the sex and gender.
Population characteristics	This study included 9 SARS-CoV-2 patients (4 females, aged between 29 and 84 years with an average of 63 years) and healthy donors. Fresh human peripheral blood leukopak were taken from healthy human donors. Certificates are available from STEMCELL TECHNOLOGIES for leukopak.
Recruitment	Patients were voluntarily enrolled in the study with written informed consent.
Ethics oversight	The research followed the protocol approved by the Institutional Review Board (IRB) at Providence St. Joseph Health with IRB study number STUDY2020000175 and the Western Institutional Review Board (WIRB) with IRB study number 20170658.

Note that full information on the approval of the study protocol must also be provided in the manuscript.

Field-specific reporting

Please select the one below that is the best fit for your research. If you are not sure, read the appropriate sections before making your selection.

- [x] Life sciences
- [ ] Behavioural & social sciences
- [ ] Ecological, evolutionary & environmental sciences

For a reference copy of the document with all sections, see nature.com/documents/nr-reporting-summary-flat.pdf

Life sciences study design

All studies must disclose on these points even when the disclosure is negative.

Sample size	Sample size has been indicated in each case.<br>COVID19 subjects (N=9)
Data exclusions	TCR sequencing data not passing the quality control in terms of the purity was excluded.
Replication	Single cell sequencing in 10x were replicated in the two lanes. All quantitative analysis in the experiments was run in triplicate. Findings were reproducible by re-running the analysis.
Randomization	Three HLA-matched COVID 19 participants per analysis were randomly selected to have a total of nine.
Blinding	Blinding was not relevant to this study due to objective outcome measures. Study design was purely observational without therapeutic intervention.

Reporting for specific materials, systems and methods

We require information from authors about some types of materials, experimental systems and methods used in many studies. Here, indicate whether each material, system or method listed is relevant to your study. If you are not sure if a list item applies to your research, read the appropriate section before selecting a response.

Materials & experimental systems

n/a	Involved in the study
☐	☒ Antibodies
☐	☒ Eukaryotic cell lines
☒	☐ Palaeontology and archaeology
☒	☐ Animals and other organisms
☒	☐ Clinical data
☒	☐ Dual use research of concern

Methods

n/a	Involved in the study
☒	☐ ChIP-seq
☐	☒ Flow cytometry
☒	☐ MRI-based neuroimaging

Antibodies

Antibodies used	The following product was used in this experiment;<br>Alexa Fluor 488 anti-human CD8 Antibody from BioLegend, catalog # 344716, clone: SK1<br>APC anti-human TCR α/β Antibody from BioLegend, catalog # 306717, clone: IP16<br>Ultra-LEAF™ Purified anti-human CD3 Antibody from BioLegend, catalog # 317325, clone: OKT3<br>Ultra-LEAF™ Purified anti-human CD28 Antibody from BioLegend, catalog # 302933, clone: CD28.2<br>Brilliant Violet 421™ anti-human IFN-γ Antibody from BioLegend, catalog # 502531, clone: 4S.B3<br>anti-TNF-α (DY210-05), anti-IFN-γ (DY285B-05), and anti-Granzyme B (DY2906-05) antibodies were included in DuoSet® ELISA kit from R&D Systems.
Validation	All antibodies used are commercially available and have been validated by the corresponding vendors, with information provided on their websites.

Eukaryotic cell lines

Policy information about cell lines and Sex and Gender in Research

Cell line source(s)	Human HEK 293T, T2, and TM-LCL cell lines were provided by Dr.Philip D. Greenberg. Expi293F Cell line was purchased from Thermo Fisher (A14635)
Authentication	None of the cell lines were authenticated.
Mycoplasma contamination	All cell lines used tested negative for mycoplasma contamination.
Commonly misidentified lines (See ICLAC register)	No commonly misidentified lines were used in the study.

Flow Cytometry

Plots

Confirm that:

☒ The axis labels state the marker and fluorochrome used (e.g. CD4-FITC).

☒ The axis scales are clearly visible. Include numbers along axes only for bottom left plot of group (a 'group' is an analysis of identical markers).

☒ All plots are contour plots with outliers or pseudocolor plots.

☒ A numerical value for number of cells or percentage (with statistics) is provided.

Methodology

Sample preparation	Surface staining for flow cytometry and FACS was performed by staining PBMCs or CD8+ T cells in 100 µL of staining buffer (0.5% BSA and 2mM EDTA in PBS) with antibodies (1 ug/mL) and pMHC-multimer (20 nM) for 10 minutes at 4C in the dark. Cells were washed and resuspended in PBS.<br>For the intracellular staining after surface staining, cells were fixed and permeabilized by fixation/permeabilization solution (BD) for 20 min. and resuspended in 100 µL of permeabilization/wash Buffer with antibodies (1 ug/mL) for 30 minutes at 4C in the dark. Cells were washed and resuspended.
Instrument	Attune NxT Flow Cytometer (Thermo Fisher) and BD FACSAria™ Fusion Cell Sorter (BD) were used.
Software	FlowJo_v10.7.1_CL (FlowJo, LLC)
Cell population abundance	The purity of the sorted cells were determined by gating the target cells stained with antibody or/and pMHC-tetramer.
Gating strategy	A lymphocyte gate was defined first from FSC-A v SSC-A. Single cell gates were then defined on FSC-H v FSC-W. Additional

gating was performed as described in figure and extended data legends for individual experiments.

☒ Tick this box to confirm that a figure exemplifying the gating strategy is provided in the Supplementary Information.
