# Supplementary material for: Large libraries of single-chain trimer peptide-MHCs enable antigen-specific CD8+ T cell discovery and analysis
Source: Commun Biol. 2023 May 16;6:528. doi: 10.1038/s42003-023-04899-8 (PMC10186326; doi:10.1038/s42003-023-04899-8)
Supplement: Supplementary file 1 — Heath_Peer Review File [file 42003_2023_4899_MOESM1_ESM.pdf]

nature portfolio

Peer Review File

Large libraries of single-chain trimer peptide-MHCs enable antigen-specific CD8+ T cell discovery and analysis

Open Access This file is licensed under a Creative Commons Attribution 4.0 International License, which permits use, sharing, adaptation, distribution and reproduction in any medium or format, as long as you give appropriate credit to the original author(s) and the source, provide a link to the Creative Commons license, and indicate if changes were made. In the cases where the authors are anonymous, such as is the case for the reports of anonymous peer reviewers, author attribution should be to 'Anonymous Referee' followed by a clear attribution to the source work. The images or other third party material in this file are included in the article's Creative Commons license, unless indicated otherwise in a credit line to the material. If material is not included in the article's Creative Commons license and your intended use is not permitted by statutory regulation or exceeds the permitted use, you will need to obtain permission directly from the copyright holder. To view a copy of this license, visit http://creativecommons.org/licenses/by/4.0/.

Reviewers' comments:

Reviewer #1 (Remarks to the Author):

Dear authors:

This article presents a high-throughput method for preparation of soluble pMHC by designing MHC class I molecules as single-chain trimers (SCT), sequentially linking the heavy chain, β2m, and the required peptide for assembling the protein by two Linker segments. To a certain extent, the preparation of this scheme possesses high throughput and is capable of producing SCTs paired with any peptide and class I HLA allele, so this method is able to rapidly obtain information on specific T cells involved in adaptive immunity from individuals with different HLA alleles against pathogenic viral strains, providing considerable value for subsequent vaccine-based in vitro therapies as well as T cell-based in vivo therapies, and the identified TCR sequences may serve as drug candidates for future TCR-T therapies. The SCT method itself is not new.

There are a number of issues that must be addressed before publication can be considered. If the following problems are well-addressed, this reviewer believes that the important contribution of this paper is important for quantifying antigen-specific T cell responses in the viral proteome.

The relevant questions are as follows：

1. It has been demonstrated that glycosylation is closely related to the assembly and folding of pMHC, and Figure.1c demonstrates that SCT can be glycosylated, but pMHC in the physiological state is presented to the cell membrane after completion of assembly and folding on the endoplasmic reticulum and with the assistance of related molecular chaperones, and the SCT in this paper adopts the form of secretory expression, whether it will have an impact on the true binding of peptides to MHC?
2. The effect of peptide sequence on the expression of SCT library is huge, which may miss the T cell information corresponding to some important peptides, because the antigenic peptide corresponding to SCT does not necessarily have better expression. How to address this?
3. In terms of antigen-specific T-cell enrichment, where is the advantage of SCT technology compared with other similar technology, such as 10×Genomics' pMHC-specific Dextramers technology?
4. What are the advantages of SCT multimers over traditional in vitro synthesized tetramers in T cell functional assays in vitro?
5. In the FACS experiments in Figure.3c, should a control be set up as irradiated DC cells isolated from PBMC without incubation with HLA-restricted peptides and then go to test the response of SCT libraries to them after co-incubation with T cells? Should a control also be set up to stain the isolated T cells with SCT prepared with a non-relevant peptide?
6. In Fgure.5b, why some DC cell-loaded peptides in the experimental group have GzB release but not TNF-α and IFN-γ release when incubated with T cells?

Reviewer #2 (Remarks to the Author):

Chour et al optimized the Single chain trimers (SCT) technology to express HLA-epitope complex for the utility of capturing and characterizing cognizant CD8 T cells. They systematically demonstrated the construct designs and different methods to validate/select the designs. At last, they demonstrated the construction of medium-scale SCT libraries of predicted SARS-CoV-2 peptides, which were used to capture and characterize CD8 T cell clones of three SARS-CoV-2 infected donors and one never-infected donor. The results demonstrate the utility of the SCT method for representing complex HLA-epitope libraries.

Major concern: the study relied on the NetMHC4.0 server to predict the SARS-CoV-2 epitopes for three patients. However, it is well known that the prediction server is far from accurate and comprehensive. It would be very informing to compare the results of identified epitopes of this study (Fig. 4b) with the previous results of global studies to identify CD8 T cell epitopes of SARS-CoV-2 (For example: Ferretti et al. PMID: 33128877, Zhang et al. PMID: 34506741). Significant overlapping would help identify common immuno-dominant epitopes.

Minor concerns:
1. Quality and resolution of some of the figures are low, for example, Fig. 2c and 2d.
2. The three groups of TCRs from the killing experiment (Supplementary Table 7) could be further analyzed to see if any properties of TCR might correlate with their functions.
3. Since the study only tested a subpopulations of SARS-CoV-2 epitopes predicted by NetMHC4.0, given the limitations of the prediction server, the title of the manuscript "CD8 T cell responses against a whole viral proteome" seems a bit exaggerated.

Reviewer #3 (Remarks to the Author):

The authors adapted single-chain trimer (SCT) technologies into a high throughput platform for pMHC library generation, showing that hundreds can be rapidly prepared across multiple Class I HLA alleles. Then they constructed SCT libraries designed to capture SARS-CoV-2 specific CD8+ T cells from COVID-19 participants and healthy donors. These technologies should enable the rapid analyses of peptide-based T cell responses.
While this research provides sounded data and outstanding technologies, the following points may improve the quality of this manuscript:

Comments:
1、The two statements don't match: in line 38: "the full SARS-CoV-2 viral proteome" and in line 91: "immunodominant epitopes across two SARS-CoV-2 protein domains"

2、In line 38 and 232, pMHC multimers comprised of 634 pMHC multimers , but A*02.01, A*24.02, and B*07.02 HLA allele scomprised 96, 51 and 33 peptides from the spike protein, respectively, and 191 peptides from the Nsp3 protein (papain-like protease, PLpro) for A*02:01 in line 239 and 814. According to supplementary Fig.1a-c and supplementary Table3-5, some SCT proteins yields were 0.00, should those SCTs be excluded。

3、 In line 96-97and 523，using a SCT plasmid template and each peptide-encoded primer, how many bases the product of extension PCR is? Methods didn't describe clearly.

4、SCT yield variations may be related to a secretion signal, such as the leader sequence of β2m or IgGκ, which one did you choose? How many most of SCT yield? XXμg/mL in culture medium?

5、In line 681, for CMVpp65 (NLVPMVATV) SCT(PE), the light red color card has the largest proportion（59.8%）. Do you think the main sequence of CDR3α and CDR3β indicated by the light red color card are the domain sequence, other sequences have little relation with this peptide CMVpp65.

6、A*02.01, A*24.02, and B*07.02 HLA allele shoud be changed to A*02:01, A*24:02, and B*07:02.

Point-by-point response to Reviewers' comments:

Reviewer #1 (Remarks to the Author):

Dear authors:
This article presents a high-throughput method for preparation of soluble pMHC by designing MHC class I molecules as single-chain trimers (SCT), sequentially linking the heavy chain, β2m, and the required peptide for assembling the protein by two Linker segments. To a certain extent, the preparation of this scheme possesses high throughput and is capable of producing SCTs paired with any peptide and class I HLA allele, so this method is able to rapidly obtain information on specific T cells involved in adaptive immunity from individuals with different HLA alleles against pathogenic viral strains, providing considerable value for subsequent vaccine-based in vitro therapies as well as T cell-based in vivo therapies, and the identified TCR sequences may serve as drug candidates for future TCR-T therapies. The SCT method itself is not new.
There are a number of issues that must be addressed before publication can be considered. If the following problems are well-addressed, this reviewer believes that the important contribution of this paper is important for quantifying antigen-specific T cell responses in the viral proteome. The relevant questions are as follows：

1. It has been demonstrated that glycosylation is closely related to the assembly and folding of pMHC, and Figure.1c demonstrates that SCT can be glycosylated, but pMHC in the physiological state is presented to the cell membrane after completion of assembly and folding on the endoplasmic reticulum and with the assistance of related molecular chaperones, and the SCT in this paper adopts the form of secretory expression, whether it will have an impact on the true binding of peptides to MHC?

The reviewer brings up an interesting point. The vast majority of SCTs that are reported in our manuscript do not contain glycsolyated peptides. Further, many of the SCT library elements are shown to capture T cells that also recognize the antigen presented by antigen-presenting cells. It is always possible that the glycosylation biochemistry of the secreted SCTs is unique relative to membrane bound pMHC, but it is also possible that our observations provide new opportunities. Glycosylated peptide antigens are a subject of research, especially within the framework of glycosylated self-antigens and autoimmunity. Whether the SCT library approach can provide a tool set for exploring this biology is an open question, but the fact that such peptides can be produced may provide opportunities for new studies. We agree with the referee that this is worth mentioning.

Changes. Discussion, paragraph 1, text with reference added: **Our finding that biologically produced SCTs can contain glycosylated antigens (Fig. 1c) may be notable. Such antigens, especially glycosylated self-antigens in the context of autoimmune disease, are a topic of recent literature[42]. Whether the SCT library technology can provide a new tool for exploring this biology is an open question**.

2. The effect of peptide sequence on the expression of SCT library is huge, which may miss the T cell information corresponding to some important peptides, because the antigenic peptide corresponding to SCT does not necessarily have better expression. How to address this?

This is an excellent point, although it is also a limitation of folded pMHCs or any other pathway towards producing pMHC multimers. We have taken two approaches to address this limitation, although we also acknowledge that this limitation remains.

The first is that we compared SCT production with what can be achieved using the dominant existing approach for preparing pMHC libraries, so that we can at least compare how our SCT libraries stack up against a literature gold standard that has been used for many biological studies. This comparison is supplied in Fig S1d, where we show that, for a 37-element library, most antigens that are (or are not) loaded using the UV exchange approach are similarly presented by SCTs. For the D8 template comparison, 13 of the peptide antigens yield neither a pMHC nor a SCT. UV exchange generates only 3 pMHCs that are not produced as SCTs, and the D8 template yields 1 SCT that is not produced by UV exchange. This strong equivalence between these two methods suggests that SCT libraries are at least very similar to those produced by UV exchange.

The second approach is that we clone and test many of the TCRs that we identify as antigen specific, which is presented in Figure 5, and show that those T cell clonotypes are stimulated when the antigen is presented in its native format.

However, it is possible, and even likely, that any approach that utilizes multimers is going to miss antigens that are naturally presented by MHC within the in vivo biological context. We now include some text in the discussion, paragraph #1, to indicate that we are aware of this limitation.

Changes: Discussion, paragraph 1, text added: **It is also possible, of course, that certain peptides that are presented by MHC in the natural, in vivo biological format, are not amenable to preparation as an SCT, although this limitation can also apply to *in vitro* folded pMHCs.**

3. In terms of antigen-specific T-cell enrichment, where is the advantage of SCT technology compared with other similar technology, such as 10×Genomics' pMHC-specific Dextramers technology?

4. What are the advantages of SCT multimers over traditional in vitro synthesized tetramers in T cell functional assays in vitro?

Here we answer queries #3 and #4 together

- SCT libraries can be extended to virtually any Class I HLA allele. This is not true of other large library methods. We have recently reported the use of SCT libraries that contain around 10^3 elements across multiple HLA alleles. That paper was primarily a report of a clinical trial, and so the SCT library tech was only mentioned, but not deeply described. In any case, this is a scale that is not readily accomplished using other methods.
- SCTs can be prepared in massively parallel fashion, and then stored (cryogenically) until ready for use. This combination of production and stability is unique to SCTs. Refolding tanks, for example, do not lend themselves to massively parallel production of pMHCs.

- SCT libraries were used to demonstrate significant efficiencies, relative to established literature approaches, in the targeted identification of antigen-specific CD8+ T cell populations (Fig 3 and text). This would not be possible with standard pMHCs because those constructs do not exhibit long-term stability.
- SCTs can be assembled into multimer formats, including the dextramer technology referred to above. Thus, we aren't competitive with that technology, but we can take advantage of it.

These advantages of the SCT library technology are already highlighted throughout the text, but we do emphasize some points with new text in the discussion..

Changes. Discussion, paragraph 3, text added: **Further, large SCT libraries across many additional HLA A, B, and C alleles were reported recently by some of us to identify TCRs that were translated into the clinic for adoptive cell transfer cancer immunotherapy[43]. That paper was primarily a clinical study that only briefly described the SCT library technique, but it does offer further evidence of the versatility of the method.**

5. In the FACS experiments in Figure.3c, should a control be set up as irradiated DC cells isolated from PBMC without incubation with HLA-restricted peptides and then go to test the response of SCT libraries to them after co-incubation with T cells?

6. Should a control also be set up to stain the isolated T cells with SCT prepared with a non-relevant peptide?

We answer these two queries together. These questions from the referee were largely answered in the protocols and experimental methods of Figure 3, but that text was poorly written and not clear. We have extensively revised the text to highlight that equivalent controls were in place.

Prior works have demonstrated that peptide-loaded DCs are necessary for specific expansion of antigen-specific T cell populations.[3,4] In the absence of peptide-loaded DCs, there would either be poor or non-specific expansion of T cells, rendering downstream steps (SCT tetramer sorting and expansion) extremely low-yield and requiring SCT reagents to have extremely high sensitivities, as the non-specific expansion step essentially dilutes the desired population of T cells. This is thus well-established in the literature, and citations have been included.

The equivalent negative and positive controls that we included in the Figure 3 Methods 1-3 involve the use of libraries of stimulating peptides and associated SCTs. Some of those peptides are relevant to the particular patient (antigen-specific t cell populations exist), and some are irrelevant. Pooled analysis of the cells tests those cells against all relevant and irrelevant peptides, thus providing for negative, positive, and selectivity controls. The fact that we see selective response to specific peptides and not to others is in alignment with foundational papers which propose the two-signal hypothesis (1: pMHC-TCR engagement, 2: IL-2) for T cell activation.[5]

Changes: **We have extensively revised the results text associated with Figure 3 (and Supplemental Figure S3).**

6. In Fgure.5b, why some DC cell-loaded peptides in the experimental group have GzB release but not TNF-α and IFN-γ release when incubated with T cells?

Such a diverse response of T-cell cytokine secretion upon stimulation is expected.

For example, similar results have been reported that CD8+ cells that induced GzB production upon the HIV peptide stimulation did not induce IFN-γ, while several HIV peptides that induced IFN-γ did not induce GzB[6]. The heterogeneous and polyfunctional response of T cells has also been reported in numerous studies indicating that the cytokine secretion profiles vary in T cells[2,7–10].

One contributing factor may be peptide dose, which was held at similar levels for all measurements reported here, but is also known to have a clear impact on the T cell response to antigen stimulation. This is likely related to TCR-pMHC binding affinity/avidity[11,12].

T cells captured by pMHC tetramers (and thus presumably SCT multimers) can exhibit a broad spectrum of the cellular responses to stimulation[13]. This may be related to the differentiation state of the T cells, which is not part of this study[14,15].

Changes: In the discussion, paragraph 4, we added the following text: **While all TCR clonotypes were selectively activated following antigen exposure, that level of activation was diverse. Such diverse responses have been previously reported[12,13,16]. Mechanistic studies have revealed that TCR-pMHC binding affinity may be one influencing factor, but that there are likely others[11,17,18].**

Reviewer #2 (Remarks to the Author):

Chour et al optimized the Single chain trimers (SCT) technology to express HLA-epitope complex for the utility of capturing and characterizing cognizant CD8 T cells. They systematically demonstrated the construct designs and different methods to validate/select the designs. At last, they demonstrated the construction of medium-scale SCT libraries of predicted SARS-CoV-2 peptides, which were used to capture and characterize CD8 T cell clones of three SARS-CoV-2 infected donors and one never-infected donor. The results demonstrate the utility of the SCT method for representing complex HLA-epitope libraries.

Major concern: the study relied on the NetMHC4.0 server to predict the SARS-CoV-2 epitopes for three patients. However, it is well known that the prediction server is far from accurate and comprehensive.

The referee makes an excellent point. As indicated, there are more comprehensive algorithm approaches reported for developing lists of putative antigens. While the NetMHC4.0 predictions were sufficient for the scientific studies reported in our manuscript, we do now provide text to acknowledge this limitation.

Changes: Discussion, paragraph 3, we added the text: **The antigens tested here were based upon those predicted by NetMHC4.0. Improved prediction approaches, based upon the use of multiple algorithms, have been reported[19], and could be adapted for more comprehensive searches of antigen-specific T cell populations**

It would be very informing to compare the results of identified epitopes of this study (Fig. 4b) with the previous results of global studies to identify CD8 T cell epitopes of SARS-CoV-2 (For example: Ferretti et al. PMID: 33128877, Zhang et al. PMID: 34506741). Significant overlapping would help identify common immuno-dominant epitopes.

Response: We have added the text in the section "SCT libraries enable rapid discovery…", paragraph 2: **In fact, certain antigen-specific T cell responses detected here (against epitopes QYIKWPWYI, NYNYLYRLF, and SPRRARSVA YLQPRTFLL, although YLQPRTFFK was found here to be more immunodominant), have been reported elsewhere[35,37–39].**

Minor concerns:
1. Quality and resolution of some of the figures are low, for example, Fig. 2c and 2d.

This has been corrected in the revision.

2. The three groups of TCRs from the killing experiment (Supplementary Table 7) could be further analyzed to see if any properties of TCR might correlate with their functions.

This is beyond the scope of the work, which is to describe the SCT library approach, and to show multiple scientific demonstrations of the value of this technology. A detailed query of T cell clonotypes with differential activation characteristics upon stimulation is beyond scope, although we do elaborate, in the discussion, on factors that may contribute to these diverse responses.

Changes: In the discussion, paragraph 4, we added the following text: **While all TCR clonotypes were selectively activated following antigen exposure, that level of activation was diverse. Such diverse responses have been previously reported[12,13,16]. Mechanistic studies have revealed that TCR-pMHC binding affinity may be one influencing factor, but that there are likely others[11,17,18].**

3. Since the study only tested a subpopulations of SARS-CoV-2 epitopes predicted by NetMHC4.0, given the limitations of the prediction server, the title of the manuscript "CD8 T cell responses against a whole viral proteome" seems a bit exaggerated.

We agree and have fixed this.

Reviewer #3 (Remarks to the Author):

The authors adapted single-chain trimer (SCT) technologies into a high throughput platform for pMHC library generation, showing that hundreds can be rapidly prepared across multiple Class I HLA alleles. Then they constructed SCT libraries designed to capture SARS-CoV-2 specific CD8+ T cells from COVID-19 participants and healthy donors. These technologies should enable the rapid analyses of peptide-based T cell responses.
While this research provides sounded data and outstanding technologies, the following points may improve the quality of this manuscript:

1、The two statements don't match: in line 38: "the full SARS-CoV-2 viral proteome" and in line 91: "immunodominant epitopes across two SARS-CoV-2 protein domains"

We have fixed this. The comment was made by the other referees as well, and we agree.

2、In line 38 and 232, pMHC multimers comprised of 634 pMHC multimers , but A*02.01, A*24.02, and B*07.02 HLA allele scomprised 96, 51 and 33 peptides from the spike protein, respectively, and 191 peptides from the Nsp3 protein (papain-like protease, PLpro) for A*02:01 in line 239 and 814. According to supplementary Fig.1a-c and supplementary Table3-5, some SCT proteins yields were 0.00, should those SCTs be excluded.

The fact that these SCTs don’t express product, and that our SCT yield closely matches the yields produced by alternative and literature proven library approaches suggests that there is value in presenting the results of non-productive SCT expressions. \

<u>No change.</u>

3. In line 96-97and 523, using a SCT plasmid template and each peptide-encoded primer, how many bases the product of extension PCR is? Methods didn’t describe clearly.

Peptide-encoded primers are designed with a fixed 16 bp region to anneal upstream of the plasmid's peptide region, followed by a 27-33 bp extension region that encodes the desired peptide (9-11 amino acids in length).

<u>Changes</u>: Under Methods, in *SCT peptide library production*, we added the following text: **Briefly, for any given peptide substitution, a peptide-encoded reverse primer (binding to the IFNα2 signal sequence upstream of peptide region) and a forward primer (binding to L1 sequence downstream of peptide region) is required. Both primers have 16 bp annealing regions (reverse primer: 5’-GCCAACAGAACAGCTG-3’, forward primer: 5’-GGTTGTGGAGGTTCTG-3’). The peptide-encoded reverse primer varies for any given peptide, while the forward primer remains fixed across all peptide elements (unless one chooses to use a different L1/HLA template plasmid). Thus, the peptide-encoded reverse primer will typically be extended by another 27-33 bp to account for insertion of a 9-11 amino acid peptide (e.g. reverse primer for insertion of the peptide YLQPRTFLL: 5’-CAGCAGGAAGGTTCTAGGCTGCAGGTAGCCAACAGAACAGCTG-3’).**

4、SCT yield variations may be related to a secretion signal, such as the leader sequence of β2m or IgGκ, which one did you choose? How many most of SCT yield? XXμg/mL in culture medium?

Román et al. showed that the IFNα2 signal sequence resulted in maximal yield of recombinant proteins in HEK293 cells versus other signal peptides (e.g. serum albumin, IgG heavy chain, luciferase)[24]. Therefore, this sequence (MALTFALLVALLVLSCKSSCSVG) was incorporated upstream of the peptide as our signal peptide. For a standardized transfection reaction (1.25 μg SCT plasmid mixed into 1.25 ml Expi293 cells @ ~2.8 M cells/ml) following manufacturer's protocol, 96 hours of transfection can yield up to 4.0 mg/ml of protein. An approximate SCT yield of 0.25 mg/ml was the threshold above which SCTs were selected for purification to be used in binding assay experiments.

<u>Changes: In the Introduction, in paragraph 3, we added the following text:</u> **Briefly, a pcDNA3.1 plasmid construct encodes the IFNα2 protein secretion signal (MALTFALLVALLVLSCKSSCSVG)[24], peptide, peptide-β2m linker (L1), β2m, β2m-HLA linker (L2), HLA, and protein purification tags, and the peptide-L1-β2m-L2-HLA construct is secreted as one protein.**

5. In line 681, for CMVpp65 (NLVPMVATV) SCT(PE), the light red color card has the largest proportion（59.8%）. Do you think the main sequence of CDR3α and CDR3β indicated by the light red color card are the domain sequence, other sequences have little relation with this peptide CMVpp65.

Regarding the main sequences of CDR3α and CDR3β (indicated by light red color), these sequences are likely a private sequence used by the PBMC donor. These sequences have similarities to existing CMV TCRs from the VDJ database that can be detected through GLIPH analysis, and are detected in both the pMHC and SCT pulldowns. This population, while of high frequency, is still just one of many. We do not think that this finding is unusual, as the CMVpp65 antigen is known to be highly immunogenic, and is known to trigger clonal expansions in CMV+ individuals.

No changes

6. A*02.01, A*24.02, and B*07.02 HLA allele should be changed to A*02:01, A*24:02, and B*07:02.

Changes: This has been corrected

References

1. Purcell, A. W., Driel, I. R. & Gleeson, P. A. Impact of glycans on T-cell tolerance to glycosylated self-antigens. *Immunol Cell Biol* 86, 574–579 (2008).

2. Foy, S. P. *et al.* Non-viral precision T cell receptor replacement for personalized cell therapy. *Nature* 1–10 (2022) doi:10.1038/s41586-022-05531-1.

3. Dauer, M. *et al.* Mature Dendritic Cells Derived from Human Monocytes Within 48 Hours: A Novel Strategy for Dendritic Cell Differentiation from Blood Precursors. *J Immunol* 170, 4069–4076 (2003).

4. Ho, W. Y., Nguyen, H. N., Wolfl, M., Kuball, J. & Greenberg, P. D. In vitro methods for generating CD8+ T-cell clones for immunotherapy from the naïve repertoire. *J Immunol Methods* 310, 40–52 (2006).

5. Lafferty, K. & Cunningham, A. A New Analysis of Allogeneic Interactions. *Aust J Exp Biol Med* 53, 27–42 (1975).

6. Nowacki, T. M. *et al.* Granzyme B production distinguishes recently activated CD8+ memory cells from resting memory cells. *Cell Immunol* 247, 36–48 (2007).

7. Spear, T. T. *et al.* Altered Peptide Ligands Impact the Diversity of Polyfunctional Phenotypes in T Cell Receptor Gene-Modified T Cells. *Mol Ther* 26, 996–1007 (2018).

8. Xue, Q. *et al.* Single-cell multiplexed cytokine profiling of CD19 CAR-T cells reveals a diverse landscape of polyfunctional antigen-specific response. *J Immunother Cancer* 5, 85 (2017).

9. Huang, J. *et al.* Targeted Co-delivery of Tumor Antigen and α-Galactosylceramide to CD141+ Dendritic Cells Induces a Potent Tumor Antigen-Specific Human CD8+ T Cell Response in Human Immune System Mice. *Front Immunol* 11, 2043 (2020).

10. Han, Q. *et al.* Polyfunctional responses by human T cells result from sequential release of cytokines. *Proc National Acad Sci* 109, 1607–1612 (2012).

11. Irving, M. *et al.* Interplay between T Cell Receptor Binding Kinetics and the Level of Cognate Peptide Presented by Major Histocompatibility Complexes Governs CD8+ T Cell Responsiveness*. *J Biol Chem* 287, 23068–23078 (2012).

12. Hebeisen, M. *et al.* Identification of Rare High-Avidity, Tumor-Reactive CD8+ T Cells by Monomeric TCR–Ligand Off-Rates Measurements on Living Cells. *Cancer Res* 75, 1983–1991 (2015).

13. Hombrink, P. *et al.* Mixed functional characteristics correlating with TCR-ligand koff-rate of MHC-tetramer reactive T cells within the naive T-cell repertoire. *Eur. J. Immunol.* 43, 3038–3050 (2013).

14. Cano-Gamez, E. *et al.* Single-cell transcriptomics identifies an effectorness gradient shaping the response of CD4+ T cells to cytokines. *Nat Commun* 11, 1801 (2020).

15. Caushi, J. X. *et al.* Transcriptional programs of neoantigen-specific TIL in anti-PD-1-treated lung cancers. *Nature* 596, 126–132 (2021).

16. Friedmann, K. S. *et al.* Interdependence of CTL and NK cell cytotoxicity against melanoma cells. *Biorxiv* 2020.06.14.150672 (2020) doi:10.1101/2020.06.14.150672.

17. Schmid, D. A. *et al.* Evidence for a TCR Affinity Threshold Delimiting Maximal CD8 T Cell Function. *J Immunol* 184, 4936–4946 (2010).

18. Williams, C. M. *et al.* Normalized Synergy Predicts That CD8 Co-Receptor Contribution to T Cell Receptor (TCR) and pMHC Binding Decreases As TCR Affinity Increases in Human Viral-Specific T Cells. *Front Immunol* 8, 894 (2017).

19. Wells, D. K. *et al.* Key Parameters of Tumor Epitope Immunogenicity Revealed Through a Consortium Approach Improve Neoantigen Prediction. *Cell* 183, 818-834.e13 (2020).

20. Ferretti, A. P. *et al.* Unbiased Screens Show CD8+ T Cells of COVID-19 Patients Recognize Shared Epitopes in SARS-CoV-2 that Largely Reside outside the Spike Protein. *Immunity* 53, 1095-1107.e3 (2020).

21. Nelde, A. *et al.* SARS-CoV-2-derived peptides define heterologous and COVID-19-induced T cell recognition. *Nat Immunol* 22, 74–85 (2021).

22. Kared, H. *et al.* SARS-CoV-2-specific CD8+ T cell responses in convalescent COVID-19 individuals. *J Clin Invest* 131, (2021).

23. Sekine, T. *et al.* Robust T Cell Immunity in Convalescent Individuals with Asymptomatic or Mild COVID-19. *Cell* 183, 158-168.e14 (2020).

24. Román, R. *et al.* Enhancing heterologous protein expression and secretion in HEK293 cells by means of combination of CMV promoter and IFNα2 signal peptide. *J Biotechnol* 239, 57–60 (2016).

REVIEWERS' COMMENTS:

Reviewer #1 (Remarks to the Author):

Dear authors:

Thanks for submitting this revision. I do not have further questions regarding this version. As a result, I recommend acceptance of the current version for publication.

Reviewer #2 (Remarks to the Author):

The authors have adequately addressed my concerns.

Reviewer #3 (Remarks to the Author):

Thanks. All my concerns about this manuscript have been well addressed.
